# Acetyl- and malonyl-CoA availability drive EPA selectivity in polyketide synthase-engineered *Yarrowia lipolytica*

**DOI:** 10.1186/s12934-026-02950-x

**Published:** 2026-03-04

**Authors:** Hang Qi, Fabian Ries, Sofija Jovanovic Gasovic, Demian Dietrich, Katja Gemperlein, Rolf Müller, Michael Kohlstedt, Christoph Wittmann

**Affiliations:** 1 Institute of Systems Biotechnology, Saarland University, Saarbrücken, Germany; 2 Department of Pharmaceutical Biotechnology, Helmholtz Institute for Pharmaceutical Research Saarland, Saarbrücken, Germany

**Keywords:** *Yarrowia lipolytica*, Polyketide synthase, ω‑3 fatty acids, EPA, DPA, DHA, Acetyl‑CoA, Malonyl‑CoA, Precursor remodeling, l-lysine supplementation, Transcriptomics, Fed‑batch fermentation

## Abstract

**Background:**

The oleaginous yeast *Yarrowia lipolytica* is an attractive chassis for sustainable production of long‑chain ω‑3 polyunsaturated fatty acids (PUFAs). Polyketide synthase (PKS)-like PUFA synthases bypass the canonical oxygen‑dependent desaturase/elongase route, yet the influence of precursor availability on PKS product selectivity in *Y. lipolytica* remains unclear.

**Results:**

Here, we explored a panel of *Y. lipolytica* strains comprising single‑origin (*Aetherobacter fasciculatus*,* Minicystis rosea*) and hybrid PKS clusters. A domain‑shuffled producer, Hyb6, broadened the product spectrum to penta‑unsaturated ω‑3 species, yielding EPA (18.3 mg L^-1^), DPA (38.8 mg L^-1^) and trace DHA (1.5 mg L^-1^) in shake flasks. Time-resolved metabolomics revealed that ω-3 accumulation began in the stationary phase, when acetyl-CoA and malonyl-CoA pools were strongly reduced. l-lysine supplementation upon glycerol depletion was associated with elevated malonyl-CoA levels, accelerated EPA formation (4.6-fold vs. control), and maintenance of an EPA/DPA ratio > 1.9. In contrast, a ketogenic amino-acid mix increased native lipids but reduced EPA selectivity. Transcriptomics revealed l-lysine‑dependent upregulation of acetyl‑CoA supply nodes (*ACL1*/*ACL2*, *ACS*, *ACC1*) and l-lysine catabolism (*KAT1*, *GCDH*, *UGA2*), together with induction of amino‑acid transporters and protein‑folding machinery. In fed‑batch processes, pulsed l-lysine selectively increased EPA to 405.5 mg L^-1^ (11.8% selectivity), with DPA at 321.5 mg L^-1^ and DHA at 14.0 mg L^-1^.

**Conclusions:**

Changes in acetyl-CoA and malonyl-CoA availability are strongly associated with EPA selectivity. Coupling modular PKS design with targeted precursor remodeling provides a versatile strategy to fine-tune product spectra in *Y. lipolytica* and related microbial PUFA cell factories.

**Supplementary Information:**

The online version contains supplementary material available at 10.1186/s12934-026-02950-x.

## Background

Long-chain omega-3 polyunsaturated fatty acids (ω‑3 PUFAs), including eicosapentaenoic acid (EPA, C20:5), docosapentaenoic acid (DPA, C22:5), and docosahexaenoic acid (DHA, C22:6), are essential nutrients that play key roles in cardiovascular, neural, and retinal health [1–5]. However, humans cannot synthesize ω‑3 PUFAs *de novo* and must obtain them from dietary sources, primarily marine fish oils [6]. The declining sustainability of marine resources has intensified efforts to establish renewable, scalable microbial production systems [7]. The oleaginous yeast *Y. lipolytica* has emerged as a robust microbial platform for the sustainable production of lipid-derived compounds [8]. It is a non-conventional yeast that is generally recognized as safe (GRAS). The organism shows high metabolic flexibility, a remarkable capacity for lipid accumulation, and efficient utilization of diverse carbon sources [9–11]. These attributes make *Y. lipolytica* an attractive host for producing high-value fatty acids (FAs), including PUFAs [12–14].

Traditional metabolic engineering of *Y. lipolytica* for ω‑3 PUFA production has primarily relied on the aerobic desaturase/elongase pathway [15]. This pathway converts C18 precursors through sequential desaturation and elongation steps into long-chain ω‑3 fatty acids, but it requires oxygen and large amounts of NAD(P)H, which can limit yield and scalability [16, 17]. An alternative, oxygen-independent strategy involves heterologous expression of polyketide synthase (PKS)-like PUFA synthases, which assemble long-chain PUFAs *de novo* from acetyl-CoA and malonyl-CoA precursors. PKS-based biosynthesis reduces cofactor demand and decouples ω-3 PUFA formation from the host’s native fatty acid metabolism, potentially allowing a more direct control over chain length and unsaturation. Recent advances have shown that *Y. lipolytica* can express PKS-like PUFA synthases from myxobacteria, enabling de novo DHA synthesis under oxygen-independent conditions [18]. This modular pathway bypasses the limitations of the classical desaturase/elongase system and allows tuning of chain domains from different bacterial origins, and domain swaps from different bacterial origins have been proposed to diversify ω-3 PUFA profiles. However, their characterization in *Y. lipolytica* is still limited. While PKS domain architecture defines the enzymatic potential for ω-3 chain elongation and termination, the intracellular availability of acetyl-CoA and malonyl-CoA may act as a key determinant of product selectivity. Recent work using isotopic tracing demonstrated that l-lysine catabolism contributes directly to acetyl-CoA supply in PKS-expressing *Y. lipolytica* [19], suggesting that precursor-level control could complement genetic design in impacting ω-3 product distributions.

Here, we investigate how PKS domain architecture and acetyl-/malonyl-CoA precursor remodeling through amino acid supplementation jointly influence ω-3 PUFA synthesis in *Y. lipolytica*. Using strains expressing single-species and hybrid PKS clusters, we identify architectures that expand the product range toward EPA and DPA. Integrated metabolomic and transcriptomic analyses reveal how l-lysine supplementation remodels CoA metabolism and central carbon flux to enhance ω-3 PUFA formation. Finally, fed-batch validation demonstrates that targeted l-lysine feeding for acetyl- and malonyl-CoA reinforcement provides a scalable strategy for tuning PKS-based ω-3 PUFA production.

## Results

### Domain-level hybridization redirects the PKS product spectrum toward EPA/DPA

**Fig. 1:**
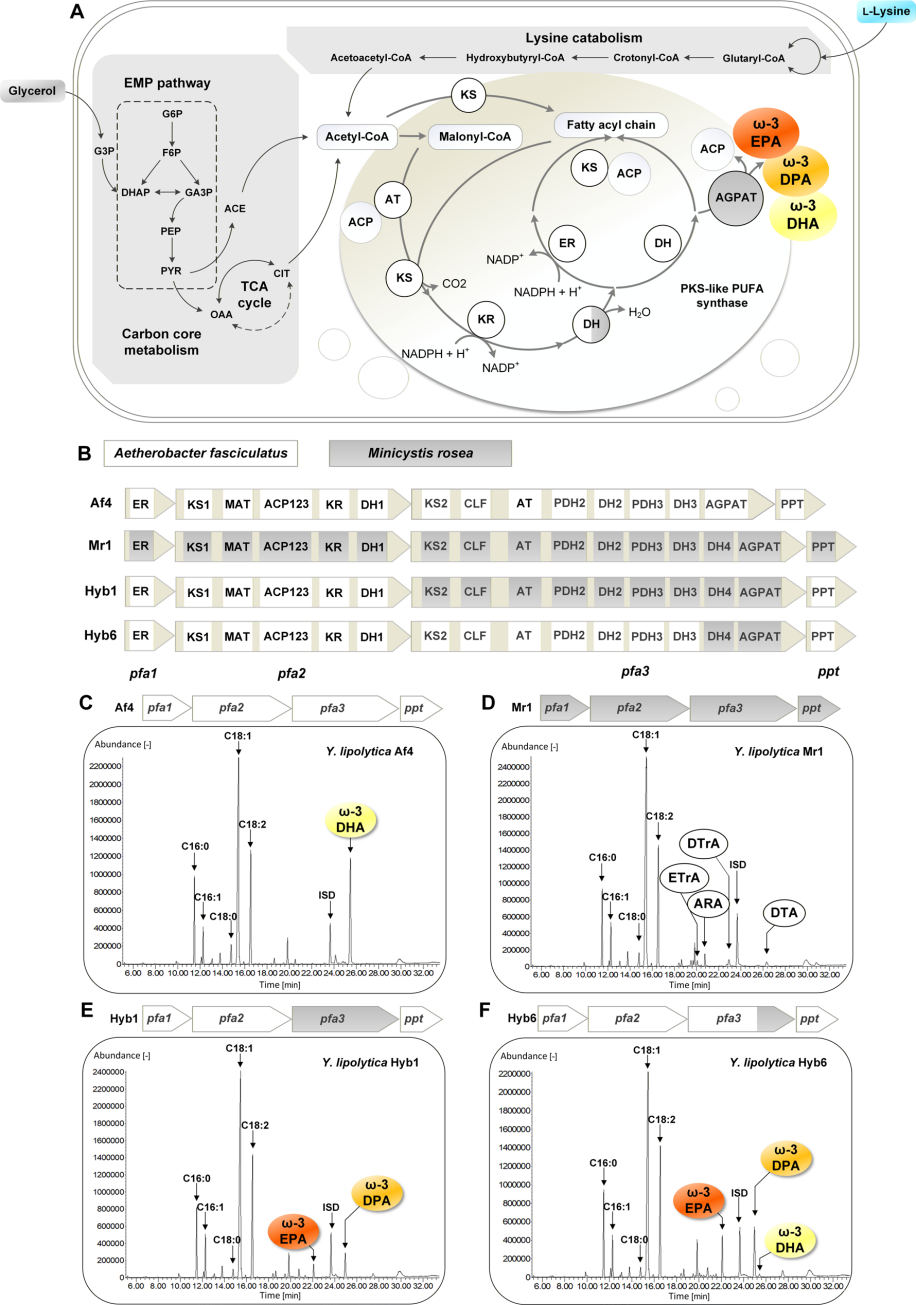
Hybrid PKS architecture determines the ω-3 PUFA product spectrum in *Y. lipolytica*.** A** Schematic representation of the PKS-based ω-3 PUFA biosynthetic pathway.** B** Schematic overview of single-origin (Af4, Mr1) and hybrid (Hyb1, Hyb6) PUFA synthase gene clusters derived from *Aetherobacter fasciculatus* and *Minicystis rosea*, highlighting domain composition and hybridization points.** C**–**F** Representative fatty acid profiles determined by GC–MS for strains Af4 (**C**), Mr1 (**D**), Hyb1 (**E**), and Hyb6 (**F**) during shake-flask cultivation, showing the distribution of ω-3 PUFAs (EPA, DPA, DHA) and native fatty acids. Hybridization of pfa3 domains (DH4 and AGPAT) redirected the product spectrum from DHA-dominant (Af4) toward EPA/DPA-dominant production (Hyb6). Data represent means ± SD of biological triplicates

We first benchmarked four PUFA synthase architectures in *Y. lipolytica*—two single-origin clusters (Af4: *Aetherobacter fasciculatus*; Mr1: *Minicystis rosea*) and two hybrids (Hyb1, Hyb6) (Fig. 1A–F). The Af4 strain, expressing the four-gene - *pfa1*, *pfa2*, *pfa3*, and *pp*t - cluster from *A. fasciculatus* as 20.2-kb genomic insert, functioned as a DHA specialist, producing 96.1 mg L^− 1^ DHA alongside endogenous C16 and C18 fatty acids (e.g. oleate 224.9 mg L^− 1^, linoleate 80.4 mg L^− 1^, palmitate 47.3 mg L^− 1^) (Fig. 1C). In contrast, strain Mr1, expressing the *M. rosea* cluster, lost DHA formation but shifted production to tri- and tetra-unsaturated ω-3/ω-6 species, albeit at low abundance (arachidonic acid (ARA) 5.1 mg L^− 1^, docosatrienoic acid (DTrA) 4.5 mg L^− 1^, docosatetraenoic acid (DTA) 3.7 mg L^− 1^, eicosatrienoic acid (ETrA) 2.7 mg L^− 1^; Fig. 1D).

Hybridization reprogrammed the PUFA spectrum. Strain Hyb1 (*pfa1*, *pfa2*, *ppt* from *A. fasciculatus* and *pfa3* from *M. rosea*) yielded penta-unsaturated ω-3 products, such as EPA (5.7 mg L^− 1^) and 14.7 mg L^− 1^ DPA as dominant species and no DHA (Fig. 1E). A more subtle domain swap in Hyb6—retaining the *A. fasciculatus* genes *pfa1*–*pfa2*–*ppt* and most of *pfa3* but replacing the *pfa3* domains DH4 and AGPAT with their *M. rosea* homologs—increased EPA to 18.3 mg L^− 1^, DPA to 38.8 mg L^− 1^, and DHA to 1.5 mg L^− 1^ (Fig. 1F). Relative to Hyb1, Hyb6 increased EPA 2.2-fold and DPA 1.6-fold while regaining DHA. These data identified *pfa3*—and specifically its encoded module DH4 and the termination module AGPAT—as key determinant for setting chain length and unsaturation balance, enabling simultaneous access to EPA and DPA in *Y. lipolytica*.

### Stationary-phase ω-3 accumulation emerges under severe CoA precursor limitation

**Fig. 2:**
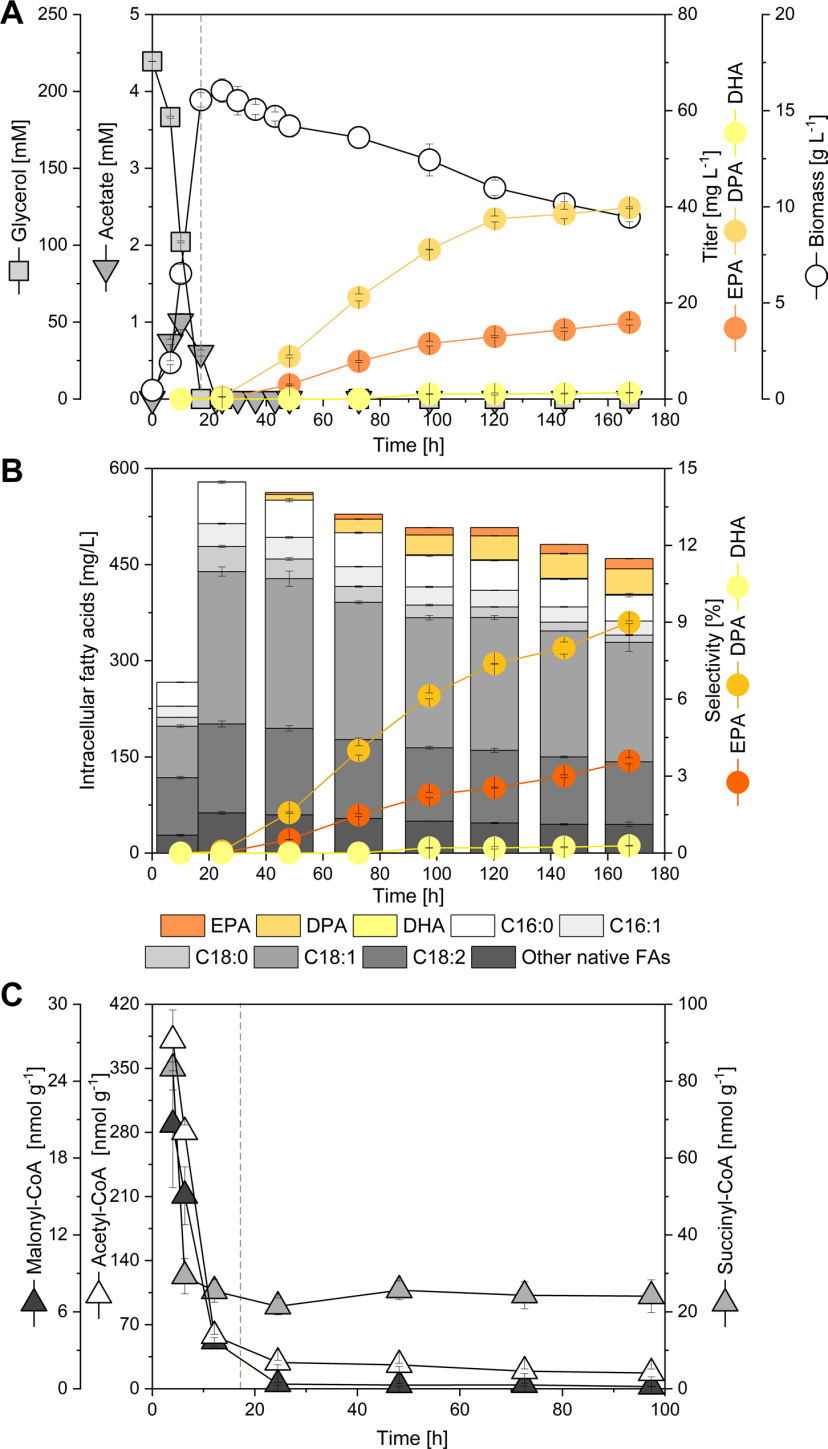
Stationary-phase ω-3 accumulation under CoA precursor limitation.** A** Growth and extracellular metabolite profiles of Hyb6 cultivated on glycerol (220 mM).** B** Time course of total native lipids and ω-3 fatty acids (DPA, EPA, DHA).** C** Intracellular acetyl-CoA and malonyl-CoA concentrations during growth and stationary phases. The onset of PKS-mediated ω-3 formation occurred after carbon depletion under severely reduced CoA pools. Dashed lines indicate the phases before and after glycerol depletion. Data represent mean ± SD (*n* = 3)

In glycerol batch cultures of Hyb6 (220 mM glycerol), growth ceased after 24 h at 16.0 g L^− 1^ biomass with transient acetate accumulation (1.0 mM), while citrate remained undetectable (Fig. 2A). Native storage lipids reached 578.6 mg L^− 1^ at the end of the growth phase (24 h; Fig. 2B), coinciding with a sharp decline in intracellular acetyl-CoA and malonyl-CoA pools from 380.5 to 28.7 nmol g^− 1^ (− 92%) and from 20.6 to 0.4 nmol g^− 1^ (− 98%), respectively (Fig. 2C). Despite this severe precursor depletion, ω-3 PUFA synthesis initiated only after carbon depletion and entry into stationary phase. DPA accumulated first at 24 h, followed by EPA at 48 h and DHA at 96 h, reaching 39.8, 15.9, and 1.3 mg L^− 1^, respectively, by 120 h. During this period, endogenous lipids declined by approximately 30%, indicating mobilization of storage pools to support PUFA synthesis.

Other CoA intermediates exhibited characteristic trends: succinyl-CoA showed an early decline followed by stabilization, reflecting preserved TCA cycle activity (Fig. 2C), whereas short-chain acyl-CoA levels from β-oxidation and amino acid catabolism (e.g., butyryl-, crotonyl-, and propionyl-CoA) declined sharply (Additional file 1: Fig. S1). Glycerol supported higher lipid and ω-3 PUFA titers than glucose and accelerated CoA pool turnover (Additional file 1: Fig. S2). Collectively, these results reveal that ω-3 fatty acid formation in Hyb6 is temporally decoupled from bulk acetyl-CoA and malonyl-CoA abundance, proceeding under a regime of pronounced acetyl-/malonyl-CoA limitation characteristic of stationary-phase metabolism.

### l-lysine supplementation enhances CoA metabolism and selectively boosts EPA formation

**Fig. 3:**
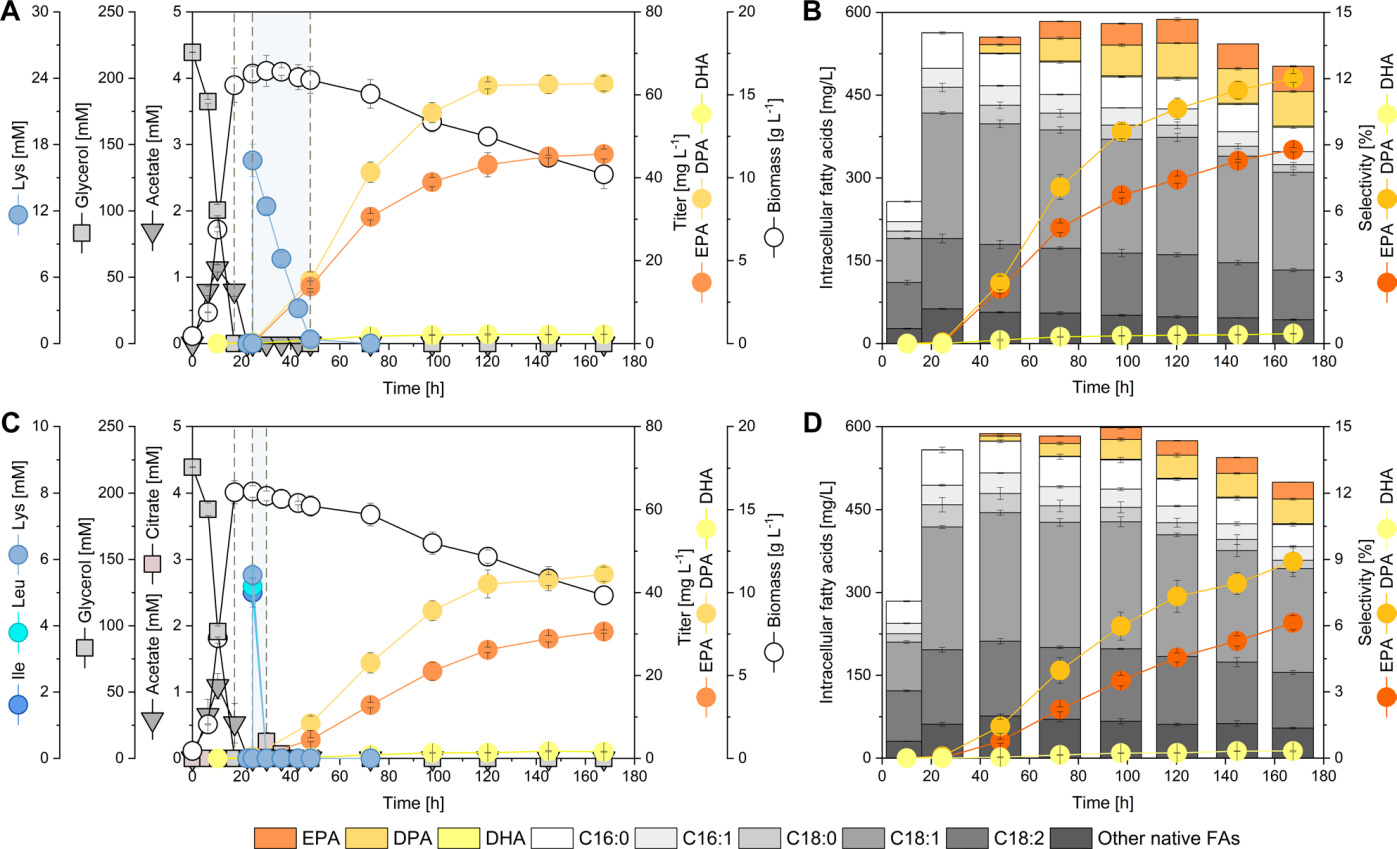
l-lysine and mixed ketogenic amino-acid supplementation alter ω-3 PUFA production.** A**,** B** ω-3 and native fatty-acid profiles after 15 mM l-lysine feeding at 24 h.** C**,** D** Corresponding data for the mixture of l-lysine, l-leucine, and l-isoleucine (5 mM each). l-lysine selectively increased EPA and DPA, whereas the mixture enhanced native lipid synthesis. The dashed lines mark the time points of glycerol depletion, amino acid addition and consumption of the latter, respectively. Values are means ± SD (*n* = 3)

Acetyl-CoA serves as a central precursor for fatty-acid and PUFA biosynthesis, and its intracellular availability can be replenished through degradation of ketogenic amino acids [20]. To explore whether this route could strengthen precursor supply and improve ω-3 PUFA synthesis, two supplementation strategies were tested: addition of 15 mM l-lysine alone or a mixture of l-lysine, l-leucine, and l-isoleucine (5 mM each) (Fig. 3). These amino acids enter central metabolism through distinct catabolic routes that converge at the acetyl-CoA pool, either directly (isoleucine) or via acetoacetyl-CoA intermediates (l-lysine, l-leucine), thereby potentially boosting flux toward lipid biosynthesis.

**Fig. 4:**
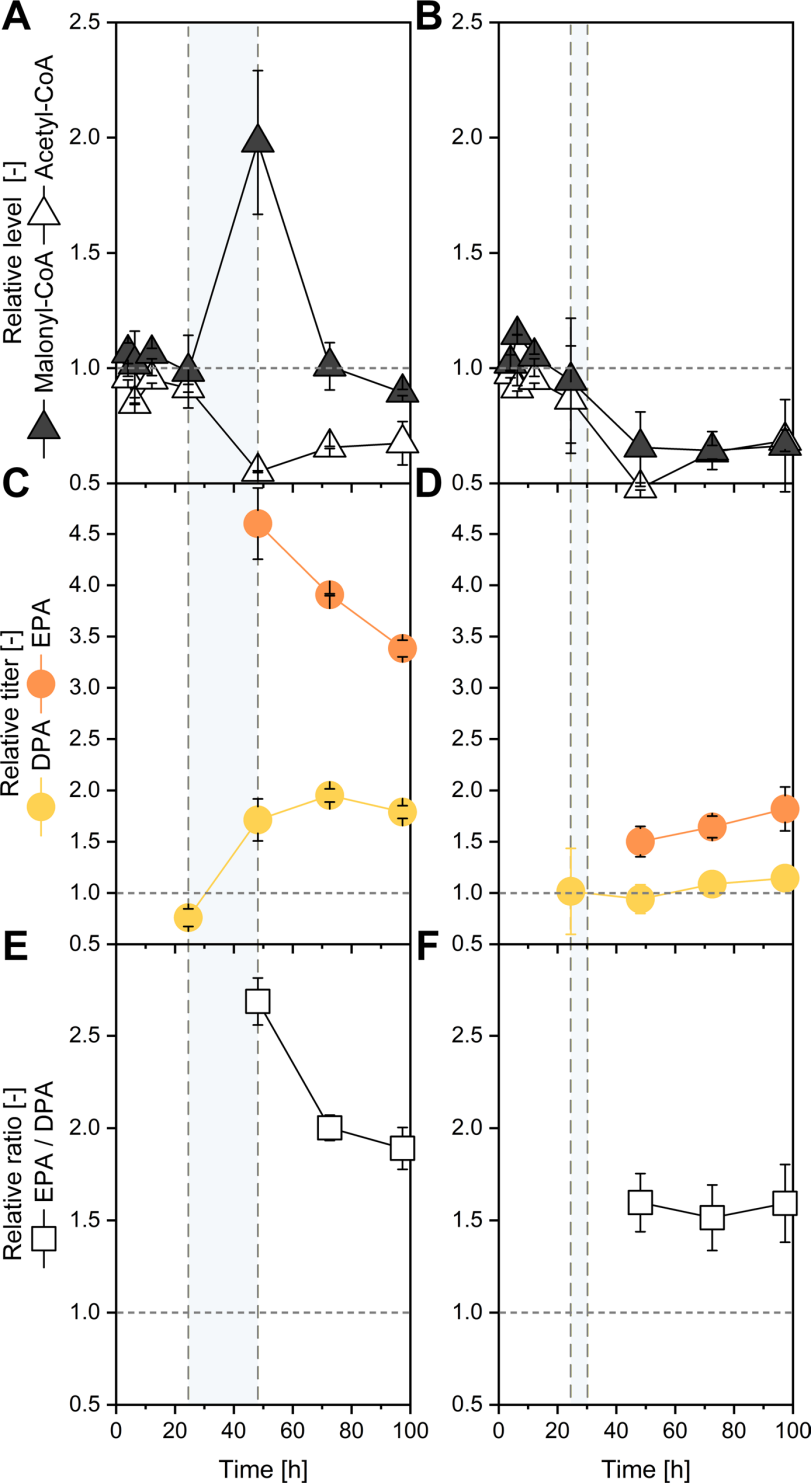
CoA precursor dynamics and EPA selectivity following amino-acid supplementation.** A**,** B** Intracellular acetyl-CoA and malonyl-CoA concentrations following supplementation with l-lysine (**A**) or a mixture of ketogenic amino acids (l-lysine, l-leucine, and l-isoleucine;** B** relative to the unsupplemented control.** C**,** E**) Time-resolved accumulation of EPA and DPA following -lysine supplementation** C** or supplementation with the ketogenic amino acid mixture (**E**).** D**,** F** Corresponding EPA/DPA ratios over time for l-lysine (**D**) and ketogenic amino acid mixture (**F**) supplementation. Relative titers are expressed as fold changes normalized to the unsupplemented control at the respective time point. Dashed lines indicate the time points of glycerol depletion, amino acid addition, and amino acid consumption. Data represent mean ± SD (*n* = 3)

Addition of l-lysine at 24 h, coinciding with glycerol depletion, caused a modest increase in biomass but produced a substantial increase in ω-3 PUFA accumulation. EPA and DPA reached 45.7 and 62.7 mg L^− 1^, respectively, while total fatty acids increased to 502.3 mg L^− 1^, approximately 9% above the unsupplemented control (Fig. 3A). The increase was mainly attributable to PUFAs, as native C16–C18 fatty acids remained nearly constant (Fig. 3B). Intracellular CoA profiling revealed a 2-fold rise in malonyl-CoA and a partial restoration of acetyl-CoA, consistent with enhanced conversion of acetyl-CoA into malonyl-CoA via acetyl-CoA carboxylase activity (Fig. 4A). EPA accumulation responded rapidly, reaching 4.6-fold of the control after 48 h, whereas DPA rose more gradually to a 2-fold maximum at 72 h (Fig. 4C). The EPA/DPA ratio peaked at 2.7 and remained above 1.9 throughout the production phase, indicating that l-lysine-associated acetyl-/malonyl-CoA availability is linked to preferential EPA formation over longer-chain products (Fig. 4D).

In contrast, supplementation with the amino-acid mixture yielded a different metabolic response (Fig. 3C-D). EPA and DPA titers increased relative to the control but were lower than with l-lysine alone, whereas native lipid accumulation rose sharply to 423.2 mg L^− 1^, the highest among all tested conditions. Here, malonyl-CoA levels declined below control values (0.7-fold of the control at 48 h), suggesting diversion of acetyl-CoA toward lipid storage rather than PKS flux. EPA reached only 1.5-fold enhancement, with a nearly constant EPA/DPA ratio of 1.5–1.6 (Fig. 4B, D, F). These results indicate that the combined presence of leucine and isoleucine redirected carbon flux from PUFA formation toward general lipid biosynthesis, likely reflecting differences in how their catabolic products feed the TCA cycle and acetyl-CoA pool [19].

Both supplementation regimes promoted mobilization of storage lipids during the stationary phase, as indicated by enhanced turnover of endogenous fatty acids (Additional file 1: Fig. S3). However, only l-lysine supplementation yielded a strong and sustained stimulation of EPA formation together with elevated malonyl-CoA levels. Considering that l-lysine catabolism proceeds through the saccharopine pathway to glutaryl-CoA and ultimately acetyl-CoA, its sustained effect may derive from temporary l-lysine storage in the vacuole followed by gradual release into the cytosol, where it is converted to acetyl-CoA and supports continuous malonyl-CoA regeneration, thereby maintaining precursor availability even after external depletion [19]. Collectively, these data demonstrate that l-lysine supplementation provides an effective and inexpensive means to support continuous malonyl-CoA regeneration, consistent with sustained precursor supply even after external depletion.

### l-lysine and ketogenic amino acids induce distinct global transcriptional programs.

**Fig. 5:**
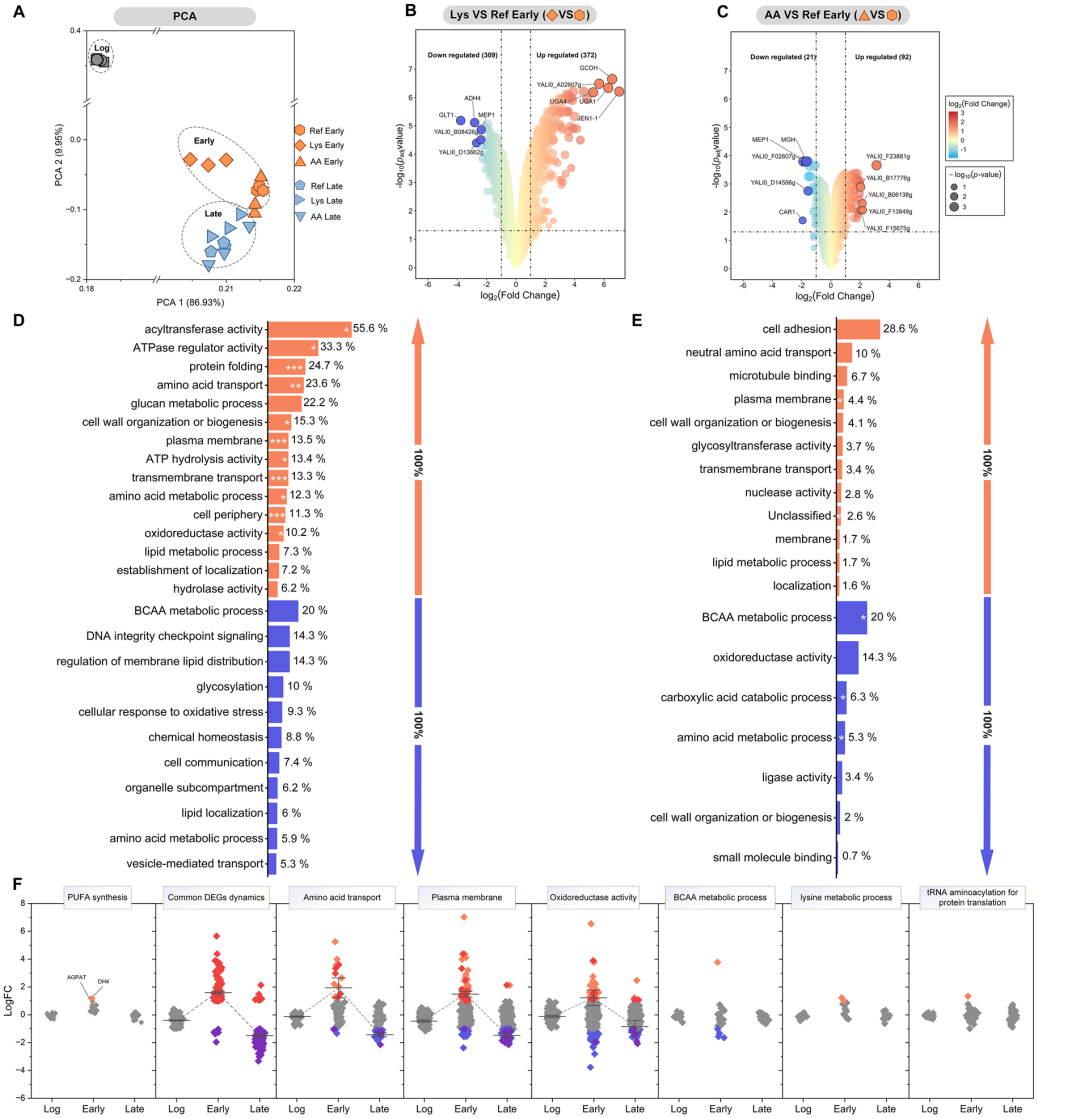
Global transcriptional responses to amino-acid supplementation.** A** Principal-component analysis (PCA) of transcriptomes showing separation by cultivation regime and phase.** B**,** C** Volcano plots of differentially expressed genes (DEGs) under l-lysine and ketogenic amino-acid supplementation (48 h).** D**,** E** Enriched Gene Ontology (GO) terms among up-regulated genes (orange) and down-regulated genes (blue).** F** Functional modules commonly affected by l-lysine supplementation. Only genes with adjusted *p* < 0.05 and |log₂FC| ≥ 1 were considered significant. Data represent mean ± SD (*n* = 3)

Global transcriptome analyses were performed for *Y. lipolytica* Hyb6 cultures under three regimes (unsupplemented, l-lysine, and ketogenic amino acid mix) at logarithmic (10 h), early production (48 h), and late production (96 h) phases. Principal component analysis (PCA) revealed clear separation by supplementation condition and cultivation stage (Fig. 5A). The first component distinguished growth from production phases, reflecting extensive reprogramming upon transition to ω-3 synthesis, while later stages showed stabilization of expression profiles.

At 48 h, l-lysine supplementation triggered a broad transcriptional activation involving 681 differentially expressed genes (372 up- and 309 downregulated; Fig. 5B). Enriched functional categories included acyltransferase and ATPase regulator activity, protein folding, and amino acid transport, consistent with intensified biosynthetic activity. Downregulated genes were mainly associated with branched-chain amino acid (BCAA) metabolism, DNA integrity checkpoint signaling, and membrane lipid distribution (Fig. 5D). In contrast, the ketogenic amino acid mixture induced a narrower response (92 up- and 21 downregulated genes; Fig. 5C), mainly affecting cell adhesion, amino acid transport, and BCAA metabolism (Fig. 5E). Gene Ontology and KEGG enrichment confirmed that l-lysine stimulated degradation of amino acids and fatty acids, whereas the mixture primarily influenced BCAA metabolism and biosynthetic pathways (Fig. 5D, E, Additional file 1: Fig. S4). Despite the global decline in transcriptional activity during the late stationary phase, 115 genes exhibited coordinated expression dynamics: being upregulated early and repressed later. They comprised modules for amino acid transport, cell wall organization, and oxidoreductase activity. (Fig. 5F, Additional file 1: Fig. S5–S6). These data indicate that l-lysine supplementation elicits a broad adaptive program to sustain metabolic balance and protein homeostasis during production phase.

### l-lysine-driven metabolic rewiring enhances acetyl-CoA supply and supports ω-3 PUFA synthesis

**Fig. 6:**
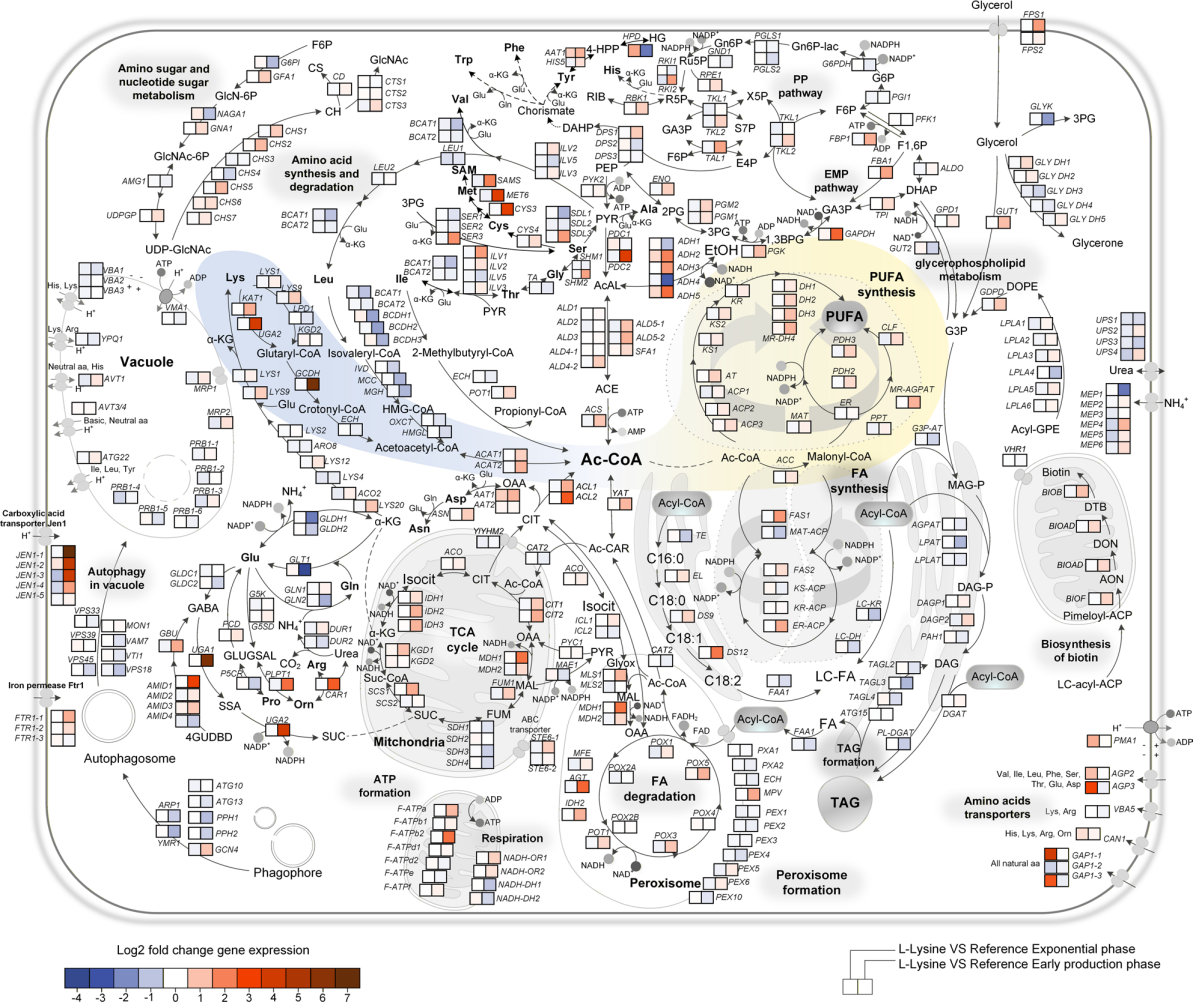
l-Lysine supplementation upregulates acetyl-/malonyl-CoA and amino-acid catabolism pathways. Expression changes of representative genes at 48 h. Upregulated genes include *ACL1/2*, *ACS*, *ACC1*, *KAT1*, *GCDH*, and *UGA2*, indicating reinforcement of acetyl-CoA generation via l-lysine degradation and citrate cycling. Data represent mean ± SD (*n* = 3)

Detailed analysis of central metabolic genes highlighted coordinated activation of acetyl-CoA generation, malonyl-CoA synthesis, and PKS-related processes (Fig. 6). At 48 h, l-lysine upregulated *ACS*, *ACL1*, *ACL2*, and *ACC1*, reinforcing acetate and citrate conversion to acetyl- and malonyl-CoA. Genes of the heterologous PKS pathway were moderately induced, while those for endogenous linoleic-acid synthesis were elevated, suggesting redirected carbon flux from storage lipids toward PUFA formation. l-lysine degradation genes (*KAT1*, *UGA2*, *GCDH*) showed strong induction, confirming l-lysine catabolism as a key acetyl-CoA source, whereas l-leucine degradation was repressed. Upregulation of *ACAT1/2* supported interconversion between acetoacetyl-CoA and acetyl-CoA. These responses collectively increase precursor availability and are consistent with sustained ω-3 synthesis during production-phase metabolism. At 96 h, expression patterns became more selective: most TCA cycle and glycolytic genes remained elevated, ensuring energy and redox balance, while nitrogen transporters were repressed, consistent with reduced assimilation. *ACC1* expression increased further, underscoring continued malonyl-CoA demand (Additional file 1: Fig. S6).

**Fig. 7:**
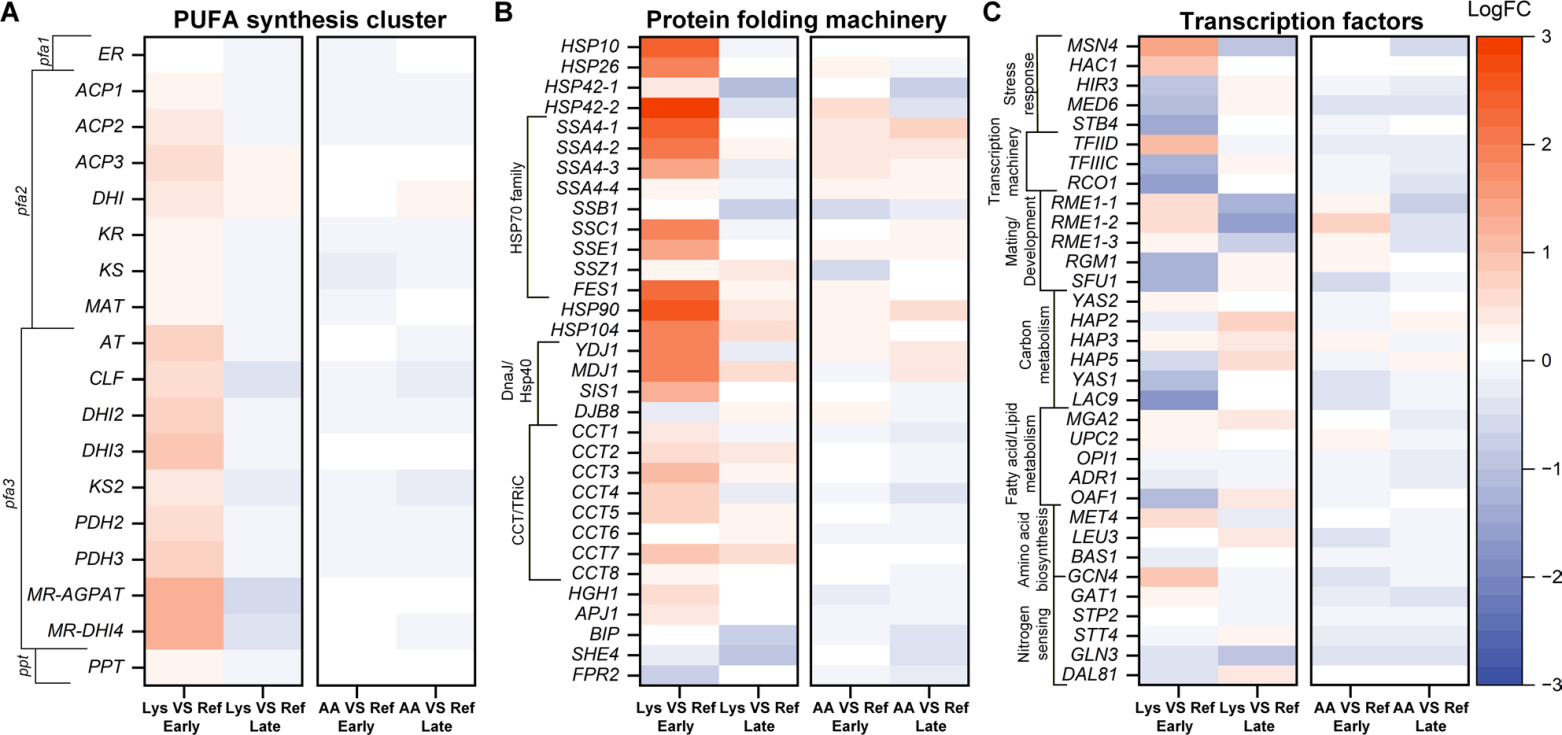
Transcriptional response of stress and regulatory networks under l-lysine feeding. Differential expression of genes encoding heat-shock proteins (HSPs), co-chaperones, and transcription factors (*GCN4*, *RCO1*, *OAF1*). Upregulation suggests enhanced protein-folding capacity and modulation of lipid-metabolic control. Data represent mean ± SD (*n* = 3)

Stage-specific regulation of amino-acid transporters (e.g., *AGP3*, *ALP1*, *GAP1-1*, *MUP1-4*) indicated strong uptake in early production and downregulation during late stationary metabolism (Additional file 1: Tables S1-S2). Chaperones and transcription factors, including *HSPs*, *GCN4*, *RCO1*, and *OAF1*, were distinctly regulated, reflecting adjustments in protein folding, stress tolerance, and lipid control networks (Fig. 7).

Collectively, these data show that l-lysine supplementation activates a coordinated transcriptional program enhancing acetyl-/malonyl-CoA metabolism and PKS flux, thereby sustaining ω-3 PUFA biosynthesis under nutrient-limited, stationary-phase conditions.

### Fed-batch process validation confirms enhanced EPA selectivity

**Fig. 8:**
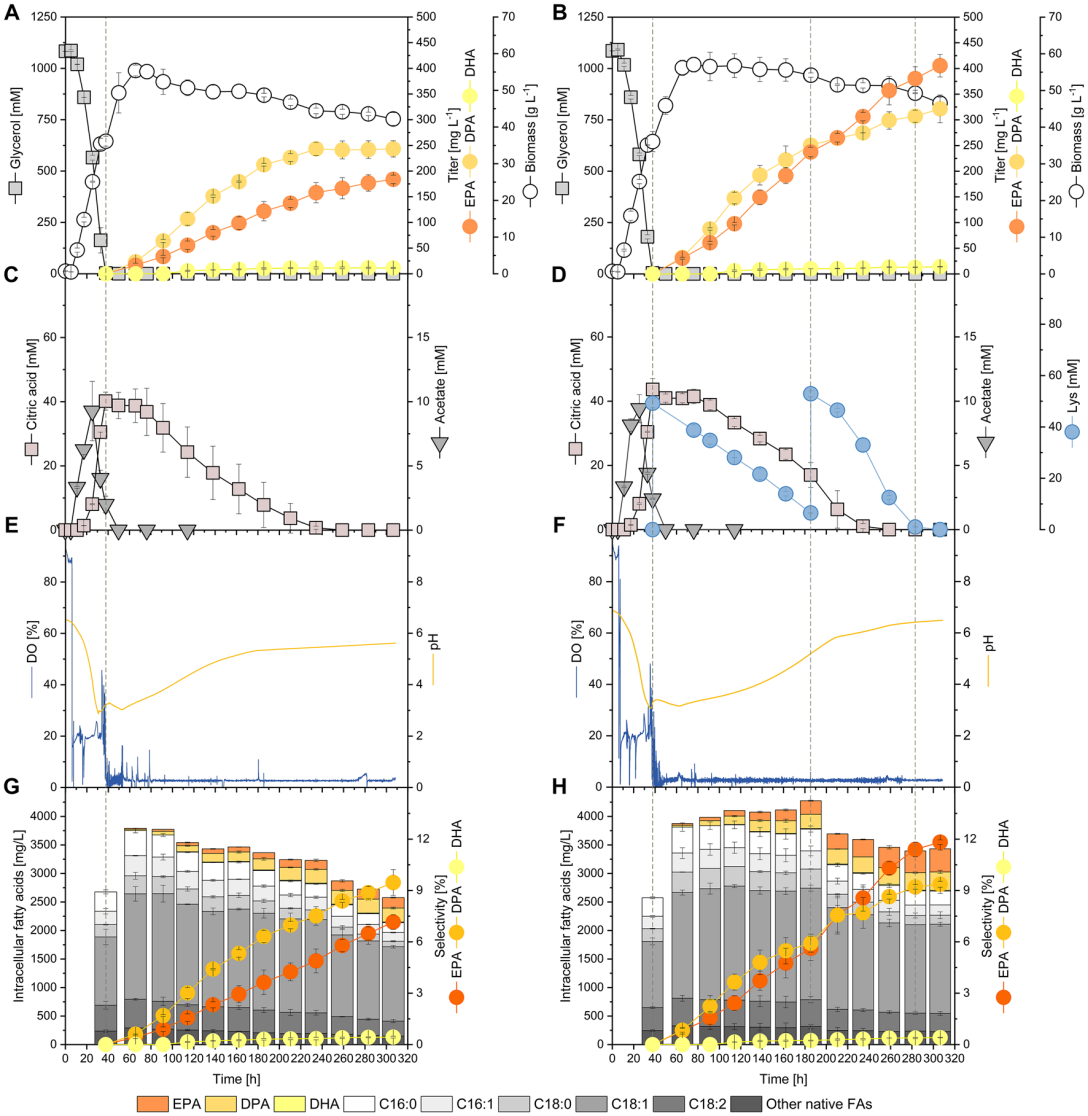
Fed-batch validation of l-lysine feeding strategy for ω-3 PUFA production.** A**,** B** Growth and glycerol consumption profiles.** C**,** D** Citrate and l-lysine uptake kinetics.** E**,** F** Dissolved oxygen (DO), agitation, and pH trends.** G**,** H** Fatty-acid composition during fermentation with and without l-lysine supplementation. l-lysine feeding maintained metabolic activity, stabilized citrate re-assimilation, and increased EPA selectivity from 7.2% to 11.8%. Values are means ± deviation (*n* = 2)

To evaluate the scalability of the l-lysine feeding strategy, we performed fed-batch fermentations of *Y. lipolytica* Hyb6 in small-scale bioreactors, comparing conditions with and without l-lysine supplementation (Fig. 8A, B). l-lysine, an inexpensive bulk chemical available from microbial fermentation of renewable raw materials [21], was supplied upon glycerol depletion (40 h post-inoculation) and again at 186 h.

During the first feeding phase, l-lysine uptake proceeded steadily, while citrate secretion persisted in both conditions (Fig. 8C, D). Notably, Hyb6 co-consumed citrate and l-lysine, suggesting that the amino acid did not inhibit organic acid re-assimilation. Upon the second l-lysine injection, uptake was markedly faster—complete consumption occurred within 100 h, indicating a potential metabolic adaptation to amino acid utilization. Process monitoring indicated distinct physiological responses between the two fermentation setups (Fig. 8E, F). Dissolved oxygen (DO) was maintained at 20% during the biomass accumulation phase and lowered to 2.5% upon entry into the stationary phase to shift host metabolism toward lipid accumulation and minimize oxidative stress, while the oxygen-independent PKS-like PUFA synthase continued ω-3 FA production. In both conditions, pH dropped sharply to ~ 3.0 during the growth phase due to organic acid formation. Thereafter, pH gradually increased during the production phase but followed distinct trajectories: in the reference process without supplementation, pH recovery required NaOH addition and stabilized at approximately 5.6, whereas in the l-lysine-supplemented culture, pH increased autonomously to 6.5. This behavior is consistent with reduced acid overflow and enhanced citrate re-assimilation specifically in the l-lysine-fed culture.

Agitation speed, which was controlled in cascade with DO, also showed pronounced differences. In the reference setup, agitation declined progressively after the growth phase, reflecting reduced oxygen uptake and metabolic activity. By contrast, in the l-lysine–supplemented fermentation, agitation remained stable at ~ 280 rpm after biomass peaked at 76 h, indicating sustained oxygen demand and metabolic activity. This elevated level persisted until 260 h, when the second l-lysine feed was nearly depleted, after which agitation dropped rapidly to less than 200 rpm, coinciding with the slowdown in substrate uptake and metabolic rate. These dynamics suggest that l-lysine supplementation not only prolonged active metabolism but also maintained higher respiratory activity well into the stationary phase, likely contributing to the extended ω-3 FA production period observed in this process.

Lipid analysis revealed that l-lysine supplementation enhanced ω-3 FA accumulation (Fig. 8G, H). In the supplemented setup, EPA titers increased continuously and, after ~ 210 h, surpassed DPA as the dominant ω-3 FA species. Final EPA selectivity reached 11.8%, significantly higher than the control (7.2%), while DPA (9.4%) and DHA (0.4%) remained comparable to the reference. Compared to the reference process, the setup with l-lysine supplementation exhibited a modest increase in both biomass concentration and native fatty acid content, and accumulated 321.5 mg L^− 1^ DPA, 405.5 mg L^− 1^ EPA, and 14.0 mg L^− 1^ DHA by the end of fermentation, representing increases of 32.2%, 120.5%, and 25%, respectively (Table 1). These results show that reinforcing acetyl-/malonyl-CoA metabolism through nutrient feeding was associated with sustained ω-3 FA biosynthesis over extended fermentation and selectively enhanced EPA accumulation, achieving higher titers and improved product profiles without impairing citrate re-assimilation or process stability. Together, these findings link PKS architecture, precursor dynamics, and nutrient signaling to ω-3 product selectivity in *Y. lipolytica*, forming the basis for rational pathway and process design.

**Table 1:** Fatty-acid composition and titers in fed-batch fermentations of *Y. lipolytica* Hyb6 with and without l-lysine supplementation

	Reference without supplementation	l-lysine supplementation
Biomass [g_CDM_ L^− 1^]	42.2 ± 1.7	46.6 ± 1.7
EPA titer [mg L^− 1^]	183.9 ± 8.2	405.5 ± 22.1
EPA selectivity [% of TFAs]	7.2 ± 0.4	11.8 ± 0.1
EPA yield [mg g_CDM_^−1^]	4.4 ± 0.4	8.7 ± 0.3
EPA productivity [mg L^− 1^ d^− 1^]	24.5 ± 1.2	51.9 ± 8.5
DPA titer [mg L^− 1^]	243.2 ± 15.9	321.5 ± 26.7
DPA selectivity [% of TFAs]	9.5 ± 0.8	9.4 ± 0.2
DPA yield [mg g_CDM_^−1^]	5.7 ± 0.6	6.9 ± 0.4
DPA productivity [mg L^− 1^ d^− 1^]	45.0 ± 11.3	63.3 ± 6.6
DHA titer [mg L^− 1^]	11.2 ± 1.1	14.0 ± 0.4
DHA selectivity [% of TFAs]	0.4 ± 0.1	0.4 ± 0.0
DHA yield [mg g_CDM_^−1^]	0.3 ± 0.1	0.3 ± 0.0
DHA productivity [mg L^− 1^ d^− 1^]	5.9 ± 0.7	6.5 ± 1.1
EPA / DPA ratio	0.8 ± 0.0	1.3 ± 0.0
Native fatty acid titer [mg L^− 1^]	2138.6 ± 64.2	2691.4 ± 164.9

Concentrations of EPA, DPA, DHA, and total fatty acids are final values (mg L^− 1^) with standard deviations (*n* = 2). EPA selectivity (%) was calculated as the mass fraction of EPA in total fatty acids at the final time point

## Discussion

### PKS pathway builds on established ω-3 biosynthesis in *Y. lipolytica*

The polyketide-synthase (PKS) route for ω-3 fatty-acid production in *Y. lipolytica* has been previously established through the heterologous expression of myxobacterial clusters [18, 19]. These works demonstrated that PKS-type PUFA synthases can function efficiently in this host, enabling oxygen-independent biosynthesis of DHA and other long-chain PUFAs. The present study builds directly on this platform and extends it toward a mechanistic understanding of domain-level control and metabolic coupling. By systematically hybridizing modules between both parental clusters, we uncovered how specific elements within *pfa3* define chain length and unsaturation balance, and how precursor economy—particularly the availability of acetyl-CoA and malonyl-CoA—modulates the resulting product profile.

### Domain logic: pfa3 as a decisive spectrum control point

Benchmarking of single-origin (Af4, Mr1) and hybrid clusters (Hyb1, Hyb6) confirmed that PKS architecture dictates the ω-3 product spectrum in *Y. lipolytica*, consistent with previous analyses in myxobacteria and thraustochytrids [18, 22–24]. The Af4 cluster operates as a DHA specialist, whereas Hyb6, incorporating the *M. rosea* DH4 and AGPAT domains within the *A. fasciculatus* framework, shifted production toward EPA/DPA with only minor DHA levels. This outcome agrees with biochemical data showing that dehydratase/isomerase modules set double-bond cadence and number, while termination domains govern chain-length release [25–27]. Thus, *pfa3*—particularly its DH4 and AGPAT modules—acts as a gatekeeper that balances elongation and termination, enabling rational tuning of PUFA chain-length distributions through modular swaps.

### Precursor economy and selective EPA formation under l-lysine feeding

Our data indicate that changes in acetyl-CoA and malonyl-CoA availability are strongly associated with PKS selectivity. Because intracellular CoA esters exhibit rapid turnover, steady-state pool sizes do not directly report metabolic flux; accordingly, the observed changes in acetyl- and malonyl-CoA levels should be interpreted as correlational indicators rather than direct measurements of pathway flux.

In addition to precursor availability, several regulatory mechanisms may contribute to the observed shift in EPA selectivity. l-lysine supplementation alters cellular nitrogen status and amino-acid signaling, which are known to influence central carbon metabolism, lipid partitioning, and stress responses in oleaginous yeasts through nutrient-sensing pathways such as TOR and related regulatory networks [28, 29]. Moreover, regulation of acetyl-CoA carboxylase (ACC1) at the post-translational level, for example via Snf1/AMPK-mediated phosphorylation, can modulate malonyl-CoA supply independently of transcript abundance and thereby decouple enzyme activity from mRNA levels [30, 31]. In parallel, the strong induction of chaperones and protein homeostasis-related genes observed under l-lysine supplementation suggests that enhanced folding capacity and stress tolerance may influence the stability and activity of the large, multienzyme PKS complex, which is structurally sensitive to cellular proteostasis [32]. While these regulatory layers were not directly interrogated in the present study, they likely act in concert with precursor remodeling to shape PKS product selectivity during stationary-phase ω-3 PUFA production.

l-lysine supplementation at the onset of stationary phase selectively increased EPA relative to DPA, raised malonyl-CoA pools, and accelerated early EPA accumulation. This pattern likely reflects kinetic competition between the C20-to-C22 elongation cycle and chain termination near C20 on the iterative PKS, consistent with structural models of β-ketoacyl-ACP synthases where elongation can become rate-limiting at high intermediate occupancy [33–36].

Mechanistically, l-lysine catabolism through the saccharopine pathway replenishes acetyl-CoA via glutaryl-CoA, while its vacuolar storage and gradual cytosolic release provide a sustained internal source of precursor even after external depletion [19]. The resulting steady acetyl-CoA supply, together with transcriptional induction of *ACC1*, supports continuous malonyl-CoA regeneration and thereby supports sustained PKS activity throughout stationary-phase production.

The delayed onset of ω-3 PUFA synthesis until carbon depletion and entry into stationary phase, despite strongly reduced acetyl-CoA and malonyl-CoA pool sizes, is a striking feature of the PKS-based pathway in *Y. lipolytica*. Similar temporal decoupling between growth and PKS activity has been reported for native PUFA-producing microorganisms, including myxobacteria and thraustochytrids, where long-chain PUFA synthesis is frequently associated with late growth or stationary-phase metabolism rather than with maximal precursor availability [22–24, 37]. In these systems, PKS expression and activity are often linked to nutrient limitation, stress responses, or developmental programs.

In the present study, this behavior may additionally be influenced by regulatory properties of the expression system. The hp4d promoter used to drive PKS expression has been reported to exhibit increased activity during late exponential and stationary phases in *Y. lipolytica* [18, 19, 38, 39], which could contribute to the observed timing of ω-3 PUFA formation. Importantly, promoter timing and precursor availability are not mutually exclusive mechanisms: late induction of PKS expression coincides with a metabolic state characterized by storage lipid mobilization and dynamic acetyl-/malonyl-CoA recycling, enabling sustained PKS activity even under low steady-state precursor pools. Together, these factors are likely to cooperate to shape the stationary-phase dominance of ω-3 PUFA synthesis observed here.

In contrast, a mixed ketogenic amino-acid feed diverted acetyl-CoA toward native lipid synthesis, lowering malonyl-CoA and reducing ω-3 selectivity—consistent with global acetyl-CoA control of lipid partitioning [40]. Together, these results demonstrate that nutrient-driven reinforcement of acetyl-/malonyl-CoA metabolism, not just enzyme composition, determines ω-3 product selectivity in PKS-expressing *Y. lipolytica*.

### Systems response: transcriptional reinforcement of precursor and energy metabolism

Transcriptome data revealed a broad l-lysine-induced upregulation of acetyl-/malonyl-CoA-related genes (*ACL1*/*ACL2*, *ACS*, *ACC1*) and l-lysine catabolic enzymes (*KAT1*, *GCDH*, *UGA2*), indicating that exogenous l-lysine fuels cytosolic acetyl-CoA through both anabolic and catabolic routes [19]. Concomitant induction of amino-acid transporters and protein-folding machinery (HSPs) suggests increased amino-acid uptake and stabilization of the large PKS complex. The coordinated activation of ACL, ACS, and ACC1 establishes a reinforced acetyl-/malonyl-CoA cycle that links precursor generation with PKS activity, ensuring sustained supply during oxygen-limited stationary metabolism. Downregulation of SDH (succinate dehydrogenase) subunits implies reduced TCA oxidative flux to limit ROS formation while maintaining precursors, a feature observed in high-DHA *Schizochytrium* strains [37]. Although canonical NADPH-generating enzymes (G6PDH, GND1, MAE1) showed little transcriptional change, *Y. lipolytica* and other oleaginous yeasts frequently regulate NADPH supply via flux control in the oxidative PPP, NADP-IDH, or ALD6 routes [41, 42]. Enhanced ACL1/ACL2 activity may further promote NADPH regeneration via citrate cycling, reinforcing reductive balance under l-lysine feeding [43, 44]. Overall, l-lysine supplementation orchestrates a coherent reconfiguration of acetyl-CoA metabolism that is consistent with sustained PKS-driven ω-3 biosynthesis under CoA-limited production-phase conditions. Although the observed transcriptional changes are consistent with increased acetyl-CoA and malonyl-CoA supply, mRNA levels do not necessarily reflect enzyme activities; therefore, the transcriptomic data are interpreted as supportive evidence for metabolic reorganization rather than as direct measures of pathway flux.

#### Process translation: prolonged metabolic activity and stable citrate re-assimilation

Fed-batch validation confirmed that l-lysine feeding maintains metabolic activity, evidenced by stable agitation at constant DO and steady pH recovery without NaOH dosing. Importantly, citrate reassimilation occurred efficiently despite continued ω-3 synthesis, alleviating a long-standing challenge in *Y. lipolytica* processes where citrate overflow competes with lipid accumulation [45–47]. The ability of l-lysine feeding to extend the productive phase can be directly linked to sustained acetyl-/malonyl-CoA cycling and delayed depletion of vacuolar l-lysine stores, which together prevent early metabolic shutdown. Compared with the non-supplemented control, l-lysine feeding increased EPA selectivity from 7.2% to 11.8% and extended the productive phase with increased specific production rates up to 0.08 without compromising process stability (Additional file 1, Fig. S7). These outcomes complement prior high-EPA desaturase/elongase processes that rely on aerobic, nitrogen-limited regimes [12, 15, 24] and demonstrate that precursor-driven PKS steering can achieve comparable selectivity under oxygen-independent conditions.

##### Integration and outlook

This study refines and expands the PKS-based ω-3 platform pioneered in *Y. lipolytica* [18, 19, 38] by elucidating how domain-level engineering and precursor control interact to govern product selectivity. The combined evidence supports a dual-axis design framework that, on the genetic axis, utilizes rational swapping of DH and AGPAT domains in *pfa3* to preset chain length and unsaturation and, on the process axis, steers metabolism through nutrient feeds (e.g., l-lysine) that elevate malonyl-CoA and modulate redox balance without increasing storage lipids. Future work might integrate dynamic l-lysine feeding, quantitative redox analysis, and CRISPRa/i-based control of acetyl-CoA and malonyl-CoA nodes to enable real-time tuning between EPA- and DPA-dominated profiles. Isotopic tracing data from PKS-expressing *Y. lipolytica* already confirm l-lysine-derived carbon incorporation into acetyl-CoA and downstream lipids [19], providing direct mechanistic evidence that precursor remodeling underlies the observed selectivity shift.

Together, these strategies illustrate how metabolic context—via acetyl-/malonyl-CoA economy—rather than enzyme identity alone—can be exploited to optimize PKS-type ω-3 biosynthesis in industrial yeast systems. The approach thus bridges molecular engineering and process design, facilitating industrial translation of PKS-based ω-3 PUFA bioprocesses.

## Conclusions

This study establishes a dual control axis for ω-3 PUFA biosynthesis in *Y. lipolytica* by integrating polyketide synthase (PKS) domain engineering with acetyl-/malonyl-CoA precursor remodeling. Hybridization of PKS modules identified pfa3 as a key element defining ω-3 product range, while nutrient-induced reinforcement of precursor metabolism enabled selective enhancement of EPA formation. Time-resolved metabolomics revealed that ω-3 synthesis initiates under severe acetyl- and malonyl-CoA limitation, and that precursor replenishment—particularly through l-lysine catabolism—reactivates PKS activity and biases the product spectrum toward EPA. Transcriptomic data supported this mechanism, showing upregulation of ACL1/ACL2, ACS, ACC1, and l-lysine-degradation genes, collectively maintaining cytosolic acetyl- and malonyl-CoA supply. The sustained effect of l-lysine feeding likely results from transient vacuolar l-lysine storage and gradual release into the cytosol, ensuring continued acetyl-CoA formation and malonyl-CoA regeneration even after external depletion. This dynamic enables prolonged PKS activity and improved product selectivity in both shake-flask and fed-batch processes. Together, these results demonstrate a strong association between acetyl- and malonyl-CoA availability and EPA selectivity in PKS-based PUFA synthesis. Coupling modular PKS design with targeted precursor control represents a generalizable strategy to steer product spectra in microbial ω-3 PUFA factories, advancing scalable and sustainable production of tailored long-chain polyunsaturated fatty acids.

## Materials and methods

### Strains and media

Prototrophic *Y. lipolytica* strains Po1h::Af4, Po1h::Mr1, Po1h::Hyb1, and Po1h::Hyb6 were used in this study [18]. These strains express heterologous PKS-like polyunsaturated fatty acid (PUFA) synthase clusters from *A. fasciculatus* (SBSr002) and *M. rosea* (SBNa008) as previously described [20]. Strains were stored as glycerol stocks (25% v/v) at − 80 °C and freshly streaked on YPD agar plates before use. For cultivation, complex yeast extract–peptone–dextrose (YPD) and defined yeast nitrogen base (YNB) media were employed. YPD medium contained per liter: 21.8 g (110 mM) glucose monohydrate (Sigma-Aldrich, St. Louis, USA), 20 g peptone, and 10 g yeast extract (both from Becton Dickinson, Heidelberg, Germany). YNB medium contained per liter: 20.3 g (220 mM) glycerol, 5 g (NH_4_)_2_SO_4_, supplements and 200 mM MES buffer (pH 6.8) [19]. The YNB base was prepared by adding all components individually [39]. Vitamins and trace elements were added as described previously [19]. For solid media, agar was added to a final concentration of 20 g L^− 1^. Amino acid supplements were filter-sterilized and added as indicated. In shake-flask experiments, either 15 mM l-lysine or a ketogenic amino acid mixture consisting of 5 mM each of l-lysine, l-leucine, and l-isoleucine was applied. In fed-batch fermentations, l-lysine was added to a final concentration of 50 mM at the specified time points.

### Shake-flask batch cultivation

Shake-flask experiments were conducted to evaluate growth, lipid accumulation, and ω-3 PUFA production under defined conditions. *Y. lipolytica* strains were first streaked on YPD agar plates and incubated at 30 °C for 24 h. Single colonies were used to inoculate 25 mL YPD precultures in 250 mL baffled Erlenmeyer flasks and cultivated at 28 °C and 230 rpm in a humidified shaker (Multitron, Infors AG, Bottmingen, Switzerland) for 8 h. Cells were harvested by centrifugation (2,500 × *g*, 3 min, 23 °C) and resuspended to inoculate 50 mL YNB precultures in 500 mL baffled flasks at an initial OD_600_ of 0.1. After overnight cultivation under the same conditions, cells were collected by centrifugation and used to inoculate 50 mL YNB main cultures at an initial OD_600_ of 1.0. Cultures were incubated at 28 °C and 230 rpm with 80% relative humidity for up to 120 h. Amino acid supplements were added as sterile-filtered solutions at the indicated time points. For l-lysine supplementation, 15 mM l-lysine was added at 24 h, corresponding to the time of glycerol depletion. For ketogenic amino acid supplementation, 5 mM each of l-lysine, l-leucine, and l-isoleucine were added under the same conditions. All cultivations were performed in biological triplicates. Samples were withdrawn at defined intervals for measurement of optical density, extracellular metabolites, fatty acid composition, intracellular CoA thioesters, and global gene expression profiles.

### Fed-batch fermentation

Fed-batch fermentations were performed in 1 L bioreactors (DASGIP SR0700ODLS, Eppendorf, Hamburg, Germany) to evaluate process performance and scalability of ω-3 PUFA production with and without amino acid supplementation. Each reactor was initially filled with 300 mL of defined production medium containing per liter: 100 g glycerol, 10 g (NH_4_)_2_SO_4_, 500 mg MgSO_4_, 1 g KH_2_PO_4_, 100 mg CaCl_2_, 100 mg NaCl, 200 µg FeCl_3_, 3 mL vitamin solution, and 3 mL trace element solution. The vitamin solution comprised (per liter): 2 mg inositol, 400 µg calcium pantothenate, 400 µg niacin, 400 µg pyridoxine hydrochloride, 400 µg thiamine hydrochloride, 200 µg p-aminobenzoic acid, 200 µg riboflavin, 2 µg biotin, and 2 µg folic acid. The trace element solution contained (per liter): 400 µg ZnSO_4_, 500 µg boric acid, 400 µg MnSO_4_, 200 µg Na_2_MoO_4_, 100 µg NaI, and 40 µg CuSO_4_. The medium was buffered with 200 mM MES (pH 6.8). Precultures were prepared as described for shake-flask experiments. Briefly, single colonies were inoculated into 25 mL YPD medium in 250 mL flasks and grown for 8 h at 28 °C and 230 rpm. Cells were then transferred to 100 mL YPD in 1 L baffled flasks at an initial OD_600_ of 0.1 and cultivated overnight. The resulting culture was harvested and used to inoculate the bioreactors. Fermentations were carried out at 28 °C ± 0.1with aeration up to 1 vvm. Dissolved oxygen (DO) was maintained at ≥ 20% during the exponential growth phase and ≥ 2.5% during the production phase by automatically adjusting agitation speed (200–800 rpm). The pH was monitored using a 405-DPAS–SC–K8S/225 probe (Mettler Toledo, Giessen, Germany) and maintained above 3.0 ± 0.1 by automatic addition of 6 M NaOH. Antifoam 204 (Sigma-Aldrich) was added manually when necessary.

Two fermentation regimes were compared: (i) a reference process without amino acid supplementation, and (ii) a process with l-lysine feeding. For the supplemented condition, l-lysine·HCl was added manually to a final concentration of 50 mM when glycerol was depleted (after about 40 h) and again when the amino acid was exhausted ( after about 186 h). Samples were taken at defined time points for analysis of biomass, extracellular metabolites, and fatty acid composition. Each condition was performed in duplicate.

### Determination of cell concentration

Cell growth was monitored by measuring the optical density at 600 nm (OD_600_) using a spectrophotometer. Cell dry mass (CDM) was calculated from OD_600_) using the correlation CDM [g L^− 1^] = 0.424 × OD_600_, as established previously [19].

### Quantification of extracellular metabolites

Samples were centrifuged (13,300 × *g*, 3 min, 4 °C), and the supernatants were analyzed by HPLC (Agilent 1200 series, Waldbronn, Germany). Glucose, glycerol, citrate, and acetate were separated on an Aminex HPX-87 H column (300 × 7.8 mm, 9 μm, Bio-Rad, Hercules, USA) operated at 45 °C with 6 mM H_2_SO_4_ as mobile phase at a flow rate of 0.5 mL min^− 1^. Compounds were detected using a refractive index detector, and concentrations were quantified by external calibration with analytical standards.

### Fatty acid analysis

Cultures containing 5 mg CDM were sampled, centrifuged (10,000 × *g*, 5 min, 4 °C), and dried in a vacuum concentrator (Savant DNA 120 SpeedVac Concentrator, Thermo Scientific, 60 min, 65 °C) [19]. Then, 15 µg of n-3 heneicosapentaenoic acid methyl ester (HPA, C21:5) (Cayman Chemical, Ann Arbor, MI, USA) was added as an internal standard. Fatty acids were extracted and converted to fatty acid methyl esters (FAMEs) using 300 µL of transesterification reagent (50% methanol, 50% toluene, 2% H_2_SO_4_) and incubated overnight at 80 °C. After cooling, 250 µL of stopping solution (0.5 M NH_4_HCO_3_, 2 M KCl) was added, vortexed, and centrifuged (10,000 × g, 5 min, room temperature). The upper organic phase containing FAMEs was analyzed by GC–MS (Agilent 6890 N, Agilent Technologies) equipped with a HP-88 column (30 m × 0.25 mm × 0.2 μm). Helium 5.0 was used as carrier gas. The temperature program started at 110 °C (1 min hold) and increased to 240 °C at 4 °C min⁻¹. Injector, transfer line, ion source, and quadrupole temperatures were set to 250, 280, 230, and 150 °C, respectively. Analytes were identified by retention time and mass spectra compared with reference standards (Supelco 37 Component FAME Mix, pure EPA and DPA methyl esters, Sigma-Aldrich). For a fatty acid species X, selectivity (%) = 100 × (mass of FA_X / total fatty acids) at the indicated time point (final harvest for Table 1).

### Amino acid quantification.

Extracellular amino acids (l-lysine, l-leucine, l-isoleucine) were determined by HPLC after pre-column derivatization with *o*-phthalaldehyde (OPA). Separation was performed on a Gemini C18 column (150 × 4.6 mm, 5 μm; Phenomenex, Aschaffenburg, Germany) at 40 °C using a gradient of 40 mM NaH_2_PO_4_ buffer (pH 7.8) and 45% acetonitrile/45% methanol/10% water at a flow rate of 1.0 mL min^− 1^ [48]. Fluorescence detection was carried out at 340 nm (excitation) and 450 nm (emission). α-Aminobutyric acid (ABU) was used as internal standard.

### Intracellular CoA thioesters

CoA thioesters were extracted according to established protocols [49]. Biomass corresponding to 8 mg CDM was quenched in 95% acetonitrile containing 25 mM formic acid (− 20 °C) and spiked with ^13^C-labeled CoA internal standards. After 10 min incubation on ice, samples were centrifuged (12,000 × *g*, 4 °C, 10 min) and the supernatants diluted with ice-cold deionized water. Combined extracts were freeze-dried, resuspended in 25 mM ammonium formate buffer (pH 3.0, 2% methanol, 4 °C), and filtered. CoA species were separated and quantified by LC–ESI–MS/MS (Agilent 1290 Infinity, QTRAP 6500+, AB Sciex, Darmstadt, Germany) using a Kinetex XB-C18 column (100 × 2.1 mm, 2.6 μm, 100 Å; Phenomenex) and a gradient of 50 mM formic acid (pH 8.1, adjusted with 25% NH_4_OH) and methanol at 0.3 mL min^− 1^: 0–7 min, 0–10% B; 7–10 min, 10–100% B; 10–11 min, 100% B; 11–12 min, 100–0% B; 12–15 min, 0% B. Detection was performed in multiple reaction monitoring (MRM) mode using previously derived ion transitions [49]. All analytical measurements were conducted with at least three biological replicates.

### RNA extraction and quality assessment

Cells were harvested from 2 mL culture samples by centrifugation (16,000 × *g*, 4 °C, 30 s) and immediately frozen in liquid nitrogen [19]. Total RNA was extracted using the RiboPure RNA Purification Kit (Thermo Fisher Scientific) according to the manufacturer’s instructions. Residual genomic DNA was removed by treatment with the TURBO DNA-free Kit (Thermo Fisher Scientific). RNA quantity and purity were determined spectrophotometrically (NanoDrop 1000, Thermo Fisher Scientific), and RNA integrity was verified using the RNA 6000 Nano Kit on an Agilent 2100 Bioanalyzer (Agilent Technologies). Only samples with RNA integrity numbers (RIN) greater than 8 were used for further analysis.

### Microarray design and hybridization

Global gene expression analysis was performed using a custom microarray (SurePrint G3 Custom GE 8 × 60 K, Agilent Technologies) [48]. The array design was generated with the eArray online tool (Agilent Technologies) based on the annotated genome of *Y. lipolytica* CLIB122 (assembly ASM252v1, CDS GCA_000002525.1) and the integrated myxobacterial PUFA cluster genes comprising 18 enzymatic domains, including MR-DH4 and MR-AGPAT from *Minicystis rosea* and the remaining domains from *Aetherobacter fasciculatus* [19]. Three 60 bp probes were designed per gene and randomly distributed on the array using Agilent SurePrint technology. Fifty nanograms of total RNA from each sample were used for reverse transcription and cRNA synthesis with the Low Input Quick Amp WT Labeling Kit (One-Color, Agilent Technologies). The resulting cRNA was purified with the RNeasy Mini Kit (Agilent Technologies) and labeled with Cy3 to a specific activity of at least 15 pmol Cy3 per µg cRNA. A total of 600 ng of labeled cRNA was hybridized to the array using the Gene Expression Hybridization Kit (Agilent Technologies). Hybridized slides were scanned with a SureScan Microarray Scanner (Agilent Technologies).

### Data processing and analysis

Raw fluorescence data were extracted and processed using the Feature Extraction Software (Agilent Technologies). Background correction, normalization, and statistical analysis were performed with the *limma* R package. Differentially expressed genes (DEGs) were identified by moderated *t*-tests with Benjamini–Hochberg false discovery rate (FDR) correction. Genes with adjusted *p*-values < 0.05 and absolute log₂ fold-change ≥ 1 were considered significantly regulated. Functional enrichment was analyzed using Gene Ontology (GO) and KEGG pathway annotations.

## Supplementary Information

Below is the link to the electronic supplementary material.

Supplementary Material 1. Additional file 1: Table S1. Log₂ fold changes of genes encoding amino acid transporters during the early and late stationary phases under l-lysine and ketogenic amino acid supplementation, relative to the control condition without supplementation. Adjusted *P*-value significance levels are indicated as follows: Table S2. Transporter orthologs in *Saccharomyces cerevisiae*, including functional descriptions and substrate specificities of the amino acid transporters listed in Table S1. Table S3. Functional description and locus tags of representative genes. Figure S1. Intracellular CoA thioester profiles during ω-3 polyunsaturated fatty acid (PUFA) production in recombinant *Y. lipolytica* Hyb6. Absolute concentrations were quantified by LC–MS/MS using ^13^C-labeled internal standards. Panels (A, B, C) show the reference cultivation without any supplementation. Panels (D, E, F) represent conditions with l-lysine supplementation, while (G, H, I) depict conditions with ketogenic amino acid supplementation. Data are presented as the mean ± standard error of the mean from three biological replicates. Figure S2. Production profiles and CoA pool dynamics in recombinant *Y. lipolytica* Hyb6 with glucose and glycerol as the sole carbon source. (A, B) Production of ω-3 PUFAs (EPA, DPA, DHA) in minimal medium with 110 mM glucose (A) or 220 mM glycerol (B) as the sole carbon source. (C–H) Intracellular CoA thioester dynamics measured over the same cultivations. (C, D) The abundance of acetyl-CoA, malonyl-CoA, and succinyl-CoA. (E, F) The abundance of butyryl-CoA, isovaleryl-CoA, and crotonyl-CoA. (G, H) The abundance of HMG-CoA, 3-hydroxybutyryl-CoA, and propionyl-CoA. (I, J) Cellular concentrations of native fatty acids and ω-3 FAs. EPA, eicosapentaenoic acid (C20:5); DPA, docosapentaenoic acid (C22:5); DHA, docosahexaenoic acid (C22:6); C18:0, stearic acid; C16:1, palmitoleic acid; C16:0, palmitic acid; C18:2, linoleic acid, C18:1, oleic acid; Other native fatty acids in low amounts, such as docosanoic acid (C22:0), tetracosanoic acid (C24:0), and hexacosanoic acid (C26:0), are given as summed fractions. The mean and standard error of three biological replicates are represented. Figure S3. Dynamics of the relative consumption rate of native fatty acids during glycerol-based cultivation. Data refers to cultures with l-lysine supplementation (A) or ketogenic amino acid supplementation (B), compared to the non-supplemented glycerol cultivation process. Data are shown as a percentage of total fatty acid consumption. Figure S4. Volcano plots comparing differential gene expression between supplemented and non-supplemented conditions during the late stationary phase. (A) llysine supplementation vs. control, (B) ketogenic amino acids supplementation vs. control. (C) Gene Ontology (GO) enrichment analysis using the Panther statistical overrepresentation tool, based on differentially expressed genes (DEGs) from (A), with an adjusted p-value < 0.05 and |log₂ fold change| ≥ 1. Plots show the proportion of DEGs in significantly enriched GO categories (orange for upregulated genes, blue for downregulated genes). Significantly enriched biological processes are indicated by asterisk (*FDR < 0.05, ** FDR < 0.01, *** FDR < 0.001). Figure S5. KEGG pathway enrichment analysis under amino acid supplementation. Differentially expressed genes (DEGs) with an adjusted p-value < 0.05 and |log₂ (fold change) | ≥ 1 were analyzed for their association with enriched pathways. Enrichment plots compare gene expression profiles between supplemented and non-supplemented conditions during the early production phase: (A) l-lysine supplementation vs. control, (B) ketogenic amino acid supplementation vs. control. Pathway ratios are calculated as GeneRatio / BgRatio, representing the proportion of DEGs within a given pathway. Significant pathway enrichment is indicated as follows: * *P* adj < 0.05, ** *P* adj < 0.01, *** *P* adj < 0.001. Figure S6. Transcriptional dynamics in central metabolism and supporting pathways of *Y. lipolytica* Hyb6 expressing a myxobacterial PKS-like synthase for PUFA production from glycerol. Gene expression was measured at three time points: exponential growth phase (10 h), early stationary phase (48 h), and late stationary phase (96 h) under l-lysine-supplemented conditions, compared to reference without supplementation. Data are the average of three biological replicates. The 350 genes analyzed represent key metabolic pathways involved in glycerol utilization, citrate and acetate metabolism, the EMP pathway, the pentose phosphate pathway, the TCA cycle, CoA ester metabolism, amino acid metabolism, lipid synthesis and breakdown, PUFA formation, and other supporting pathways. Full gene lists and raw data are available at the Gene Expression Omnibus (GEO). Abbreviations: 1,3BPG: 1,3-Bisphosphoglyceric acid; 2PG: 2-Phosphoglycerate; 3PG: 3-Phosphoglycerate; 4GUDBD: 4-Guanidinobutanamide; 4GUDBUTN: 4-Guanidinobutanoate; 4HPP: 4-Hydroxyphenylpyruvate; AcAL: Acetaldehyde; Ac-CAR: Acetyl-carnitine; ACE: Acetate; Acyl-GPE: Acyl-sn-glycero-3-phosphoethanolamine; AON: 8-Amino-7-oxononanoate; CIT: Citrate; CH: Chitin; CS: Chitosan; DAG: Diacylglycerol; DAG-P: Diacylglycerol phosphate; DAHP: 2-Dehydro-3-deoxy-D-arabino-heptonate 7-phosphate; DHAP: Dihydroxyacetone phosphate; DON: 7,8-Diamino-nonanoate; DOPE: sn-Glycerol-3-phosphoethanolamine; DTB: Dethiobiotin; E4P: Erythrose 4-phosphate; F1,6P: Fructose 1,6-bisphosphate; F6P: Fructose 6-phosphate; FUM: Fumarate; G3P: Glycerol-3-phosphate; G6P: Glucose 6-phosphate; GA3P: Glyceraldehyde 3-phosphate; GABA: γ-Aminobutyric acid; GlcNAc: N-Acetylglucosamine; GlcN-6P: Glucosamine-6-phosphate; GlcNAc-6P: N-Acetylglucosamine-6-phosphate; GLUGSAL: L-Glutamate 5-semialdehyde; Gn6P: Gluconate 6-phosphate; Gn6P-lac: D-Glucono-1,5-lactone 6-phosphate; HG: Homogentisate; Isocit: Isocitrate; LC-FA: Long-chain fatty acids; MAG-P: Monoacylglycerol phosphate; MAL: Malate; OAA: Oxaloacetate; PEP: Phosphoenolpyruvate; PYR: Pyruvate; R5P: Ribose 5-phosphate; RIB: Ribose; Ru5P: Ribulose 5-phosphate; S7P: Sedoheptulose 7-phosphate; SSA: Succinate semialdehyde; SUC: Succinate; TAG: Triacylglycerol; UDP-GlcNAc: Uridine 5′-diphospho-N-acetylglucosamine; X5P: Xylulose 5-phosphate; α-KG: α-Ketoglutarate. Figure S7. Correlation between EPA/DPA ratio and specific product formation rate (q_p_) during fed-batch fermentations. The EPA/DPA ratio is shown on the x-axis, with corresponding q values on the y-axis. Both reference and l-lysine-supplemented conditions from Fig. 8 are represented. The data highlight the relationship between the EPA/DPA ratio and product yield during the fermentation process. Data represent mean ± deviation (*n* = 2).

## Data Availability

All data are included in this article and its supplementary files. The transcriptome dataset has been deposited in the NCBI Gene Expression Omnibus (GEO) under accession number GSE310359.
