# Supplementary material for: Acetyl- and malonyl-CoA availability drive EPA selectivity in polyketide synthase-engineered Yarrowia lipolytica
Source: Microb Cell Fact. 2026 Mar 4;25:88. doi: 10.1186/s12934-026-02950-x (PMC13063552; doi:10.1186/s12934-026-02950-x)
Supplement: Supplementary file 1 — Supplementary Material 1. Additional file 1: Table S1. Log₂ fold changes of genes encoding amino acid transporters during the early and late stationary phases under l-lysine and ketogenic amino acid supplementation, relative to the control condition without supplementation. Adjusted P-value significance levels are indicated as follows: Table S2. Transporter orthologs in Saccharomyces cerevisiae, including functional descriptions and substrate specificities of the amino acid transporters listed in Table S1. Table S3. Functional description and locus tags of representative genes. Figure S1. Intracellular CoA thioester profiles during ω-3 polyunsaturated fatty acid (PUFA) production in recombinant Y. lipolytica Hyb6. Absolute concentrations were quantified by LC–MS/MS using 13C-labeled internal standards. Panels (A, B, C) show the reference cultivation without any supplementation. Panels (D, E, F) represent conditions with l-lysine supplementation, while (G, H, I) depict conditions with ketogenic amino acid supplementation. Data are presented as the mean ± standard error of the mean from three biological replicates. Figure S2. Production profiles and CoA pool dynamics in recombinant Y. lipolytica Hyb6 with glucose and glycerol as the sole carbon source. (A, B) Production of ω-3 PUFAs (EPA, DPA, DHA) in minimal medium with 110 mM glucose (A) or 220 mM glycerol (B) as the sole carbon source. (C–H) Intracellular CoA thioester dynamics measured over the same cultivations. (C, D) The abundance of acetyl-CoA, malonyl-CoA, and succinyl-CoA. (E, F) The abundance of butyryl-CoA, isovaleryl-CoA, and crotonyl-CoA. (G, H) The abundance of HMG-CoA, 3-hydroxybutyryl-CoA, and propionyl-CoA. (I, J) Cellular concentrations of native fatty acids and ω-3 FAs. EPA, eicosapentaenoic acid (C20:5); DPA, docosapentaenoic acid (C22:5); DHA, docosahexaenoic acid (C22:6); C18:0, stearic acid; C16:1, palmitoleic acid; C16:0, palmitic acid; C18:2, linoleic acid, C18:1, oleic acid; Other native fat [file 12934_2026_2950_MOESM1_ESM.docx]

**Additional file 1 to**

**Acetyl- and malonyl-CoA availability drive EPA selectivity in polyketide synthase-engineered *Yarrowia lipolytica***

### Hang Qi^1^, Fabian Ries^1^, Sofija Jovanovic Gasovic^1^, Demian Dietrich^1^, Katja Gemperlein^2^, Rolf Müller^2^, Michael Kohlstedt^1^, and Christoph Wittmann^1#^

### ^1^Institute of Systems Biotechnology, Saarland University, Saarbrücken, Germany

### ^2^Department of Pharmaceutical Biotechnology, Helmholtz Institute for Pharmaceutical Research Saarland, Saarbrücken, Germany

### ^#^ Phone/FAX: +49-681-302-71970/71972, E-mail: christoph.wittmann@uni-saarland.de

**Table S1. Log₂ fold changes of genes encoding amino acid transporters during the early and late stationary phases under L-lysine and ketogenic amino acid supplementation, relative to the control condition without supplementation.** Data represent mean ± SD (n = 3).

Adjusted *P*-value significance levels are indicated as follows:

| **Family** | **Gene Symbol** | **Locus tag** | **LysVSRef Early** | **LysVSRef Late** | **AAVSRef Early** | **AAVSRef Late** |
| --- | --- | --- | --- | --- | --- | --- |
| **Plasma membrane transporters** | **APC (amino acid-polyamine-organocation) superfamily YAT** | | | | |  |
|  | *AGP2* | YALI0C07678g | 0.99*** | 0.60*** | -0.31** | -0.04 |
|  | *AGP3* | YALI0E20713g | 2.97*** | -1.30** | 0.48** | -0.76 |
|  | *PUT4* | YALI0D15422g | 0.79 | -0.95*** | 0.94** | 0.23 |
|  | *ALP1* | YALI0F01254g | 3.60*** | -1.20*** | 0.17 | -0.59 |
|  | *CAN1* | YALI0B19338g | 0.49** | -0.07 | -0.53* | 0.21 |
|  | *DIP5-1* | YALI0B20064g | -1.33*** | 0.44*** | -0.50** | 0.06 |
|  | *DIP5-2* | YALI0C00451g | 2.22*** | 0.76** | -0.05 | 0.48** |
|  | *GAP1-1* | YALI0B16522g | 3.99*** | 0.30 | 0.67* | -0.06 |
|  | *GAP1-2* | YALI0B19492g | -0.75* | -0.35*** | 0.07 | 0.09 |
|  | *GAP1-3* | YALI0B19800g | 2.66*** | -0.08 | 0.39 | 0.13 |
|  | *GAP1-4* | YALI0C09889g | -0.10 | -0.89*** | 0.05 | -0.47*** |
|  | *GAP1-5* | YALI0C17237g | -1.03** | -1.19*** | -0.92** | -0.39* |
|  | *GAP1-6* | YALI0E10219g | 0.53 | -1.45*** | 0.63** | -0.51** |
|  | *GAP1-7* | YALI0F19866g | 1.51** | -1.43*** | 0.71** | -0.71** |
|  | **APC (amino acid-polyamine-organocation) superfamily** | | | |  |  |
|  | *MUP1-1* | YALI0D16137g | 0.92* | -1.84*** | 0.88** | -0.45** |
|  | *MUP1-2* | YALI0F03498g | 0.52** | 0.06 | -0.58** | -0.45* |
|  | *MUP1-3* | YALI0F07018g | -0.35* | -0.31** | 0.03 | -0.56** |
|  | *MUP1-4* | YALI0F25795g | 3.34*** | -2.14*** | 1.39** | -0.32* |
|  | *DAL5* | YALI0F16423g | 1.01* | -1.16*** | 0.85* | -0.58** |
|  | *VBA5* | YALI0F28017g | 0.02 | -0.31* | -0.09* | -0.25 |
| **Vacuolar transporter** | **MFS** |  |  |  |  |  |
|  | *VBA1* | YALI0E18095g | -0.49** | 0.05 | -0.28* | -0.12 |
|  | *VBA2* | YALI0D20196g | 0.00 | -0.96*** | 0.33* | -0.24* |
|  | *VBA3* | YALI0F28017g | 0.02 | -0.31* | -0.09* | -0.25 |
|  | *ATG22* | YALI0C03608g | 0.29* | 0.01 | 0.12* | -0.12 |
|  | **AAAP** |  |  |  |  |  |
|  | *AVT1* | YALI0F30063g | 0.39 | -0.66** | 0.22* | -0.16 |
|  | *AVT3/4* | YALI0E10637g | 0.09 | 0.10 | 0.02* | -0.28* |
|  | *AVT6/7* | YALI0D11836g | -0.74* | 0.22* | -0.49* | -0.29* |
|  | **LCT** |  |  |  |  |  |
|  | *ERS1* | YALI0C15345g | -0.68** | -0.21* | 0.08* | -0.22 |
|  | *YPQ1* | YALI0F05060g | -0.33* | -0.18 | -0.21* | -0.11 |
|  | *YPQ2* | YALI0D02046g | -0.48* | 0.24 | -0.16 | 0.12 |
|  | *YPQ3* | YALI0F14949g | -0.62** | -0.33* | -0.51** | -0.53* |
| **Mitochondrial transporter** | **MC** |  |  |  |  |  |
|  | *HEM25* | YALI0E09284g | 0.34 | -1.16*** | 0.37* | -0.56*** |
|  | *ORT1* | YALI0D06798g | -0.30 | -0.33* | -0.08 | -0.28 |
|  | *SAM5* | YALI0C19195g | 0.53** | 0.45*** | -0.13 | -0.02 |
| **Other** | **APC** |  |  |  |  |  |
|  | *UGA4-1* | YALI0A03135g | 0.58 | -1.52*** | 0.28 | -0.88** |
|  | *UGA4-2* | YALI0A07579g | 0.24* | -1.08*** | 0.39** | -0.40** |
|  | *UGA4-3* | YALI0A19558g | 1.26** | -1.33*** | 0.54** | -0.29** |
|  | *UGA4-4* | YALI0D00495g | 0.19 | -1.22*** | 0.20* | -0.42** |
|  | *UGA4-5* | YALI0D17688g | 0.40 | -0.40** | 0.29** | -0.38* |
|  | *UGA4-6* | YALI0E07205g | 5.26*** | -0.81*** | 0.69** | -0.38* |
|  | *UGA4-7* | YALI0F01078g | 0.13 | -0.96*** | 0.32** | -0.44*** |
|  | **MFS** |  |  |  |  |  |
|  | *AQR1* | YALI0E10483g | 1.09*** | -0.09 | 0.04 | -0.19 |
| **H+-transporting P-type ATPase** | *PMA1* | YALI0B22066g | 1.27*** | -0.05 | 0.15 | -0.26* |
|  | *VMA1* | YALI0A09900g | -0.34* | 0.00 | 0.05 | -0.11 |

**Table S2. Transporter orthologs in *Saccharomyces cerevisiae*, including functional descriptions and substrate specificities of the amino acid transporters listed in Table S1.**

| **Gene Symbol** | **Ortholog in *S.c*** | **Functional description** | **substrate specificity** |
| --- | --- | --- | --- |
| **Plasma membrane transporters** | | | |
| **APC (amino acid-polyamine-organocation) superfamily YAT** | | |  |
| *AGP2* | YBR132c | General amino acid permease | Val, Ile, Leu, Phe, Thr |
| *AGP3* | YFL055w | General amino acid permease | Val, Ile, Leu, Phe, Ser, Thr, Glu, Asp |
| *PUT4* | YOR348c | High-affinity proline permease | Pro, GABA, Ala, Gly |
| *ALP1* | YNL270c | Arginine permease | Arg |
| *CAN1* | YEL063c | Arginine permease | His, Lys, Arg, Orn |
| *DIP5-1* | YPL265w | Dicarboxylic amino acid permease | Ala, Gly, Ser, Gln, Asn, Glu, Asp |
| *DIP5-2* | YPL265w | Dicarboxylic amino acid permease | Ala, Gly, Ser, Gln, Asn, Glu, Asp |
| *GAP1-1* | YKR039w | General amino acid permease | All natural aa |
| *GAP1-2* | YKR039w | General amino acid permease | All natural aa |
| *GAP1-3* | YKR039w | General amino acid permease | All natural aa |
| *GAP1-4* | YKR039w | General amino acid permease | All natural aa |
| *GAP1-5* | YKR039w | General amino acid permease | All natural aa |
| *GAP1-6* | YKR039w | General amino acid permease | All natural aa |
| *GAP1-7* | YKR039w | General amino acid permease | All natural aa |
| **APC (amino acid-polyamine-organocation) superfamily** | | |  |
| *MUP1-1* | YGR055w | High-affinity methionine permease | Met, Cys |
| *MUP1-2* | YGR055w | High-affinity methionine permease | Met, Cys |
| *MUP1-3* | YGR055w | High-affinity methionine permease | Met, Cys |
| *MUP1-4* | YGR055w | High-affinity methionine permease | Met, Cys |
| *DAL5* | YJR152w | Allantonate permease | Cys |
| *VBA5* | YKR105c | Basic amino acid transporter | Lys, Arg |
| **Vacuolar transporter** | | | |
| **MFS** |  |  |  |
| *VBA1* | YMR088c | Vacuolar basic Amino acid transporter | His, Lys |
| *VBA2* | YBR293w | Vacuolar basic Amino acid transporter | His, Lys, Arg |
| *VBA3* | YCL069w | Vacuolar basic Amino acid transporter | His, Lys |
| *ATG22* | YCL038c | Efflux of amino acids during autophagic body breakdown in vacuole | Ile, Leu, Tyr |
| **AAAP** |  |  |  |
| *AVT1* | YJR001w | neutral amino acid transporter | Neutral aa, His |
| *AVT3/4* | YKL146w | neutral amino acid transporter | Basic, Neutral aa |
| *AVT6/7* | YBL089W | amino acid permease | Neutral aa |
| **LCT** |  |  |  |
| *ERS1* | YCR075C | Vacuolar cystine transport | Cys |
| *YPQ1* | YOL092w | cationic amino acids transporter | Lys, Arg |
| *YPQ2* | YDR352w | cationic amino acids transporter | Arg |
| *YPQ3* | YBR147w | cationic amino acids transporter | His |
| **Mitochondrial transporter** | | | |
| **MC** |  |  |  |
| *HEM25* | YDL119c | Mitochondrial glycine transporter | Gly |
| *ORT1* | YOR130c | Mitochondrial ornithine transporter | Lys, Arg, Orn |
| *SAM5* | YNL003c | Mitochondrial S-adenosylmethionine transporter | S-Adenosylmethionine, S-adenosylhomocysteine |
| **Other** |  |  |  |
| **APC** |  |  |  |
| *UGA4-1* | YDL210w | GABA-specific permease | GABA |
| *UGA4-2* | YDL210w | GABA-specific permease | GABA |
| *UGA4-3* | YDL210w | GABA-specific permease | GABA |
| *UGA4-4* | YDL210w | GABA-specific permease | GABA |
| *UGA4-5* | YDL210w | GABA-specific permease | GABA |
| *UGA4-6* | YDL210w | GABA-specific permease | GABA |
| *UGA4-7* | YDL210w | GABA-specific permease | GABA |
| **MFS** |  |  |  |
| *AQR1* | YNL065w | Internal-membrane transporter involved in excretion of amino acids | Ala, Glu, Asp |
| **H+-transporting P-type ATPase** | | | |
| *PMA1* | YGL008c | H+-transporting P-type ATPase | H^+^ |
| *VMA1* | YDL185W | Vacuolar Membrane ATPase | H^+^ |

**Table S3. Functional description and locus tags of representative genes.**

| **Biosynthesis of amino acids** | | |
| --- | --- | --- |
| **Gene symbol** | **Functional description** | **Locus tag** |
| *GAPDH* | Glyceraldehyde 3-phosphate dehydrogenase | YALI0_C06369g |
| *RKI2* | Ribose 5-phosphate isomerase B | YALI0_F01628g |
| *HIS1* | ATP phosphoribosyltransferase | YALI0_C05170g |
| *HIS2* | Histidinol phosphatase | YALI0_E05049g |
| *HIS5* | Histidinol-phosphate aminotransferase | YALI0_E01254g |
| *SDL1* | l-Serine dehydratase | YALI0_E10307g |
| *SDL2* | Serine/Threonine dehydratase | YALI0_D02497g |
| *SDL3* | l-Serine/ l-Threonine deaminase | YALI0_B16214g |
| *TAL1* | Transaldolase | YALI0_F15587g |
| *PGK* | Phosphoglycerate kinase | YALI0_D12400g |
| *AK* | l-Aspartate 4-P-transferase | YALI0_D11704g |
| *SER1* | Phosphoserine transaminase | YALI0_F06468g |
| *SER2* | Phosphoserine phosphatase | YALI0_B20438g |
| *SER3* | 3-Phosphoglycerate dehydrogenase | YALI0_F09966g |
| *CAR1* | Arginase | YALI0_E07535g |
| *SAM1* | S-Adenosylmethionine synthetase | YALI0_B14509g |
| *FBA1* | Fructose-bisphosphate aldolase | YALI0_E26004g |
| *HOM* | Homoserine dehydrogenase | YALI0_D01089g |
| *CYS3* | Cystathionine gamma-lyase | YALI0_F05874g |
| *CYS4* | Cystathionine beta-synthase | YALI0_E09108g |
| *MET6* | Homocysteine methyltransferase | YALI0_E12683g |
| *IDH3* | Mitochondrial isocitrate dehydrogenase (NAD^+^) subunit 1 | YALI0_E05137g |
| *IDH2* | Isocitrate dehydrogenase | YALI0_F04095g |
| *SHM1* | Mitochondrial serine hydroxymethyltransferase | YALI0_D22484g |
| *SHM2* | Cytosolic serine hydroxymethyltransferase | YALI0_E16346g |
| *CIT2* | Mitochondrial citrate-synthase | YALI0_E02684g |
| *ARO1* | Pentafunctional AROM polypeptide | YALI0_F12639g |
| *ARO2* | Chorismate synthase | YALI0_D17930g |
| *ARO8* | Aromatic amino acid aminotransferase I | YALI0_E20977g |
| *DAHPS1* | Phospho-2-dehydro-3-deoxyheptonate aldolase 1 | YALI0_B22440g |
| *ARO3* | Phospho-2-dehydro-3-deoxyheptonate aldolase, phenylalanine-inhibited | YALI0_B20020g |
| *ARO4* | Phospho-2-dehydro-3-deoxyheptonate aldolase, tyrosine-inhibited | YALI0_C06952g |
| *LEU1* | 3-Isopropylmalate dehydratase | YALI0_B01364g |
| *LEU2* | 3-isopropylmalate dehydrogenase | YALI0_C00407g |
| *ASN* | Asparagine synthetase (glutamine-hydrolyzing) | YALI0_A13387g |
|  |  |  |
| **Glucogenic amino acid and ketogenic amino acid degradation** | | |
| **Gene symbol** | **Functional description** | **Locus tag** |
| *AAT1* | Aspartate aminotransferase | YALI0_F29337g |
| *AAT2* | Aspartate aminotransferase | YALI0_B02178g |
| *GLT1* | Glutamate synthase (NAD^+^) | YALI0_B19998g |
| *DAO1* | D-amino acid oxidase | YALI0_E20735g |
| *DAO2* | D-amino acid oxidase | YALI0_F10197g |
| *DAO3* | D-amino acid oxidase | YALI0_F30591g |
| *DAO4* | D-amino acid oxidase | YALI0_F31427g |
| *CYS3* | Cystathionine gamma-lyase | YALI0_F05874g |
| *CYS4* | Cystathionine beta-synthase | YALI0_E09108g |
| *AGX1* | Alanine-glyoxylate transaminase | YALI0_E16643g |
| *GAL1* | L-Glutamine amonia ligase | YALI0_E09493g |
| *GAL2* | L-Glutamine amonia ligase | YALI0_D07370g |
| *GLDC1* | Glutamate decarboxylase | YALI0_C16753g |
| *GLDC2* | Glutamate decarboxylase | YALI0_F08415g |
| *GLDH1* | NADP-specific glutamate dehydrogenase | YALI0_F17820g |
| *GLDH2* | NAD-specific glutamate dehydrogenase | YALI0_E09603g |
| *GLN1* | Glutamine synthetase | YALI0_F00506g |
| *GLN2* | Glutamine synthetase | YALI0_D13024g |
| *GFA1* | Glucosamine-fructose-6-phosphate aminotransferase | YALI0_B21428g |
| *PUT2* | Mitochondrial delta-1-pyrroline-5-carboxylate dehydrogenase | YALI0_B09647g |
| *UGA1* | 4-aminobutyrate aminotransferase | YALI0_E18238g |
| *UGA2* | Succinate-semialdehyde dehydrogenase (NADP^+^) | YALI0_F26191g |
| *KAT1* | Lysine acetyltransferase | YALI0_E05533g |
| *LYS1* | Saccharopine dehydrogenase (NAD^+^) | YALI0_B15444g |
| *LYS9* | Saccharopine dehydrogenase (NADP^+^) | YALI0_D22891g |
| *LPD1* | Dihydrolipoamide dehydrogenase | YALI0_D20768g |
| *KGD1* | 2-oxoglutarate dehydrogenase complex E1 | YALI0_E33517g |
| *KGD2* | 2-oxoglutarate dehydrogenase complex E2 | YALI0_E16929g |
| *ECH* | Enoyl-CoA hydratase | YALI0_B10406g |
| *GCDH* | Glutaryl-CoA dehydrogenase | YALI0_F23749g |
| *BCDH1* | 2- oxoisovalerate dehydrogenase subunit alpha | YALI0_D08690g |
| *BCDH2* | 2- oxoisovalerate dehydrogenase subunit beta | YALI0_F05038g |
| *BCDH3* | Lipoamide acyltransferase component of branched-chain alpha-keto acid dehydrogenase complex | YALI0_D23815g |
| *BCAT1* | Mitochondrial branched chain amino acid aminotransferase | YALI0_D01265g |
| *BCAT2* | Cytosolic branched chain amino acid aminotransferase | YALI0_F19910g |
| *ACAT1* | Acetyl-CoA C-acetyltransferase 1 | YALI0_E11099g |
| *ACAT2* | Acetyl-CoA C-acetyltransferase 2 | YALI0_B08536g |
| *IVD* | Isovaleryl-CoA dehydrogenase | YALI0_E12573g |
| *MCC* | Urea amidolyase | YALI0_B14619g |
| *OXCT* | 3-Oxoacid CoA-transferase | YALI0_F26587g |
| *HMGL* | hydroxymethylglutaryl-CoA lyase | YALI0_B22550g |
| **PUFA synthesis cluster** | | |
| **Gene symbol** | **Functional description** | **Gene ID** |
| *ER* | PKS-like enoylreductase | Pfa1 |
| *ACP1* | PKS-like acyl carrier protein 1 | Pfa2_ACP1 |
| *ACP2* | PKS-like acyl carrier protein 2 | Pfa2_ACP2 |
| *ACP3* | PKS-like acyl carrier protein 3 | Pfa2_ACP3 |
| *DHI* | PKS-like dehydratase/isomerase | Pfa2_DH1 |
| *KR* | PKS-like ketoreductase | Pfa2_KR |
| *KS* | PKS-like ketosynthase 1 | Pfa2_KS1 |
| *MAT* | PKS-like acyltransferase | Pfa2_MAT |
| *AT* | PKS-like acyltransferase | Pfa3_AT |
| *CLF* | PKS-like chain length factor | Pfa3_CLF |
| *DHI2* | PKS-like dehydratase/isomerase 2 | Pfa3_DH2 |
| *DHI3* | PKS-like dehydratase/isomerase 3 | Pfa3_DH3 |
| *KS2* | PKS-like ketosynthase | Pfa3_KS2 |
| *PDH2* | PKS-like pseudo dehydratase 2 | Pfa3_Pseudo_DH2 |
| *PDH3* | PKS-like pseudo dehydratase 3 | Pfa3_Pseudo_DH3 |
| *MR-AGPAT* | PKS-like 1-acylglycerol-3-phospatate acyltransferase | MR-AGPAT |
| *MR-DHI4* | PKS-like dehydratase/isomerase 4 | MR-DH4 |
| *PPT* | PKS-like 4′-phosphopantetheinyl transferase | ppt |

| **Protein folding machinery** | | |
| --- | --- | --- |
| **Gene symbol** | **Functional description** | **Locus tag** |
| \| *HSP10* \| \| --- \| \| *HSP26* \| \| *HSP42-1* \| \| *HSP42-2* \| \| *SSA4-1* \| \| *SSA4-2* \| \| *SSA4-3* \| \| *SSA4-4* \| \| *SSB1* \| \| *SSC1* \| \| *SSE1 SSZ1* \| \| *FES1* \| \| *HSP90* \| \| *HSP104* \| \| *YDJ1* \| \|  \| \| *MDJ1* \| \| *SIS1* \| \| *DJB8* \| \| *CCT1* \| \| *CCT2* \| \| *CCT3* \| \| *CCT4* \| \| *CCT5* \| \| *CCT6* \| \| *CCT7* \| \| *CCT8* \| \| *HGH1* \| \| *APJ1* \| \| *BIP* \| \| *SHE4* \| \| *FPR2* \| | \| 10 kDa heat shock protein 10 \| \| --- \| \| 22.7 kDa class IV heat shock protein 26 \| \| Heat shock protein 42 \| \| Heat shock protein 42 \| \| Heat shock protein 70 Ssa4 \| \| Heat shock protein 70 Ssa4 \| \| Heat shock protein 70 Ssa4 \| \| Heat shock protein 70 Ssa4 \| \| Ribosome-associated molecular chaperone Ssb1 \| \| Heat shock protein Ssc1 \| \| Heat shock protein Sse1 \| \| Ribosome-associated complex subunit Ssz1 \| \| Hsp70 nucleotide exchange factor Fes1 \| \| Heat shock protein 90 \| \| Heat shock protein 104 \| \| Dnaj-like-2 chaperone, mitochondrial and ER import protein Ydj1 \| \| Dnaj homolog 1, mitochondrial-related chaperone \| \| Dnaj homolog subfamily B member 5 chaperone \| \| DnaJ subfamily B member 8 chaperone \| \| T-complex protein 1 subunit alpha \| \| T-complex protein 1 subunit beta \| \| T-complex protein 1 subunit gamma \| \| T-complex protein 1 subunit delta \| \| T-complex protein 1 subunit epsilon \| \| T-complex protein 1 subunit zeta \| \| Putative T-complex protein 1 subunit eta \| \| T-complex protein 1 subunit theta \| \| Protein Hgh1 Homolog \| \| Putative J domain-containing protein \| \| Endoplasmic reticulum chaperone BiP \| \| Ring assembly protein 3 \| \| FK506-binding protein 2 \| | \| YALI0_B05610g \| \| --- \| \| YALI0_C03465g \| \| YALI0_E18546g \| \| YALI0_C03443g \| \| YALI0_F25289g \| \| YALI0_D08184g \| \| YALI0_E35046g \| \| YALI0_D22352g \| \| YALI0_A00132g \| \| YALI0_C17347g \| \| YALI0_E13255g \| \| YALI0_B12474g \| \| YALI0_F11121g \| \| YALI0_C07953g \| \| YALI0_E27962g \| \| YALI0_F00880g \| \| YALI0_F12551g \| \| YALI0_E13508g \| \| YALI0_F20614g \| \| YALI0_B15774g \| \| YALI0_F11473g \| \| YALI0_D20570g \| \| YALI0_F20416g \| \| YALI0_E21692g \| \| YALI0_D20328g \| \| YALI0_C20999g \| \| YALI0_D11220g \| \| YALI0_F07315g \| \| YALI0_D02002g \| \| YALI0_E13706g \| \| YALI0_E21758g \| \| YALI0_A19602g \| |

| **Transcription factors** | | |
| --- | --- | --- |
| **Gene symbol** | **Functional description** | **Locus tag** |
| \| *MSN4* \| \| --- \| \| *HAC1* \| \| *HIR3* \| \| *MED6* \| \| *STB4* \| \| *TFIID* \| \| *TFIIIC* \| \| *RCO1* \| \| *RME1-1* \| \| *RME1-2* \| \| *RME1-3* \| \| *RGM1* \| \| *SFU1* \| \| *YAS2* \| \| *HAP2* \| \| *HAP3* \| \| *HAP5* \| \| *YAS1* \| \| *LAC9* \| \| *MGA2* \| \| *UPC2* \| \| *OPI1* \| \| *ADR1* \| \| *OAF1* \| \| *MET4* \| \| *LEU3* \| \| *BAS1* \| \| *GCN4* \| \| *GAT1* \| \| *STP2* \| \| *STT4* \| \| *GLN3* \| \| *DAL81* \| | \| C2H2 zinc finger stress activator \| \| --- \| \| ER stress-responsive transcription factor \| \| Histone transcription repressor \| \| Mediator complex subunit MED6 \| \| Zinc finger transcriptional regulator \| \| TATA-binding general TF for initiation \| \| RNA Pol III initiation factor \| \| Rpd3L chromatin regulator \| \| Rme1-like developmental TF \| \| Rme1-like developmental TF \| \| Rme1-like developmental TF \| \| Pheromone-responsive TF (GRM-type) \| \| Iron homeostasis transcriptional repressor \| \| Alkane-responsive bHLH transcription factor \| \| CCAAT-binding respiration regulator \| \| CCAAT-binding respiration regulator \| \| CCAAT-binding respiration regulator \| \| Alkane-responsive bHLH transcription factor \| \| GAL4-like carbon source TF \| \| ER lipid sensor, regulates OLE1 \| \| Sterol uptake regulator \| \| Inositol-responsive phospholipid repressor \| \| Ethanol/fatty acid utilization activator \| \| Oleate-activated transcription factor \| \| Sulfur amino acid regulator \| \| Leucine biosynthesis regulator \| \| Purine/amino acid flux regulator \| \| Amino acid starvation master regulator \| \| NCR-responsive transcription factor \| \| SPS pathway amino acid sensor \| \| PI4-kinase, SPS signaling activator \| \| NCR master activator \| \| SPS component transcription activator \| | \| YALI0_C13750g \| \| --- \| \| YALI0_B12716g \| \| YALI0_D11506g \| \| YALI0_E32373g \| \| YALI0_D14872g \| \| YALI0_B23056g \| \| YALI0_C18117g \| \| YALI0_E12991g \| \| YALI0_A12925g \| \| YALI0_F15543g \| \| YALI0_E14971g \| \| YALI0_C06842g \| \| YALI0_E05555g \| \| YALI0_E32417g \| \| YALI0_F23111g \| \| YALI0_F17072g \| \| YALI0_B03322g \| \| YALI0_C02387g \| \| YALI0_D20460g \| \| YALI0_B12342g \| \| YALI0_B15818g \| \| YALI0_C14784g \| \| YALI0_D18678g \| \| YALI0_F13321g \| \| YALI0_D04466g \| \| YALI0_D13904g \| \| YALI0_B13992g \| \| YALI0_E27742g \| \| YALI0_F17886g \| \| YALI0_E24937g \| \| YALI0_E28153g \| \| YALI0_D20482g \| \| YALI0_D02805g \| |

**Figure S1. Intracellular CoA thioester profiles during ω-3 polyunsaturated fatty acid (PUFA) production in recombinant *Y. lipolytica* Hyb6.** Absolute concentrations were quantified by LC–MS/MS using ^13^C-labeled internal standards. Panels (A, B, C) show the reference cultivation without any supplementation. Panels (D, E, F) represent conditions with l-lysine supplementation, while (G, H, I) depict conditions with ketogenic amino acid supplementation. Data are presented as the mean ± standard error of the mean from three biological replicates.

**S2. Production profiles and CoA pool dynamics in recombinant *Y. lipolytica* Hyb6 with glucose and glycerol as the sole carbon source.** (A, B) Production of ω-3 PUFAs (EPA, DPA, DHA) in minimal medium with 110 mM glucose (A) or 220 mM glycerol (B) as the sole carbon source. (C–H) Intracellular CoA thioester dynamics measured over the same cultivations. (C, D) The abundance of acetyl-CoA, malonyl-CoA, and succinyl-CoA. (E, F) The abundance of butyryl-CoA, isovaleryl-CoA, and crotonyl-CoA. (G, H) The abundance of HMG-CoA, 3-hydroxybutyryl-CoA, and propionyl-CoA. (I, J) Cellular concentrations of native fatty acids and ω-3 FAs. EPA, eicosapentaenoic acid (C20:5); DPA, docosapentaenoic acid (C22:5); DHA, docosahexaenoic acid (C22:6); C18:0, stearic acid; C16:1, palmitoleic acid; C16:0, palmitic acid; C18:2, linoleic acid, C18:1, oleic acid; Other native fatty acids in low amounts, such as docosanoic acid (C22:0), tetracosanoic acid (C24:0), and hexacosanoic acid (C26:0), are given as summed fractions. The mean and standard error of three biological replicates are represented.

###
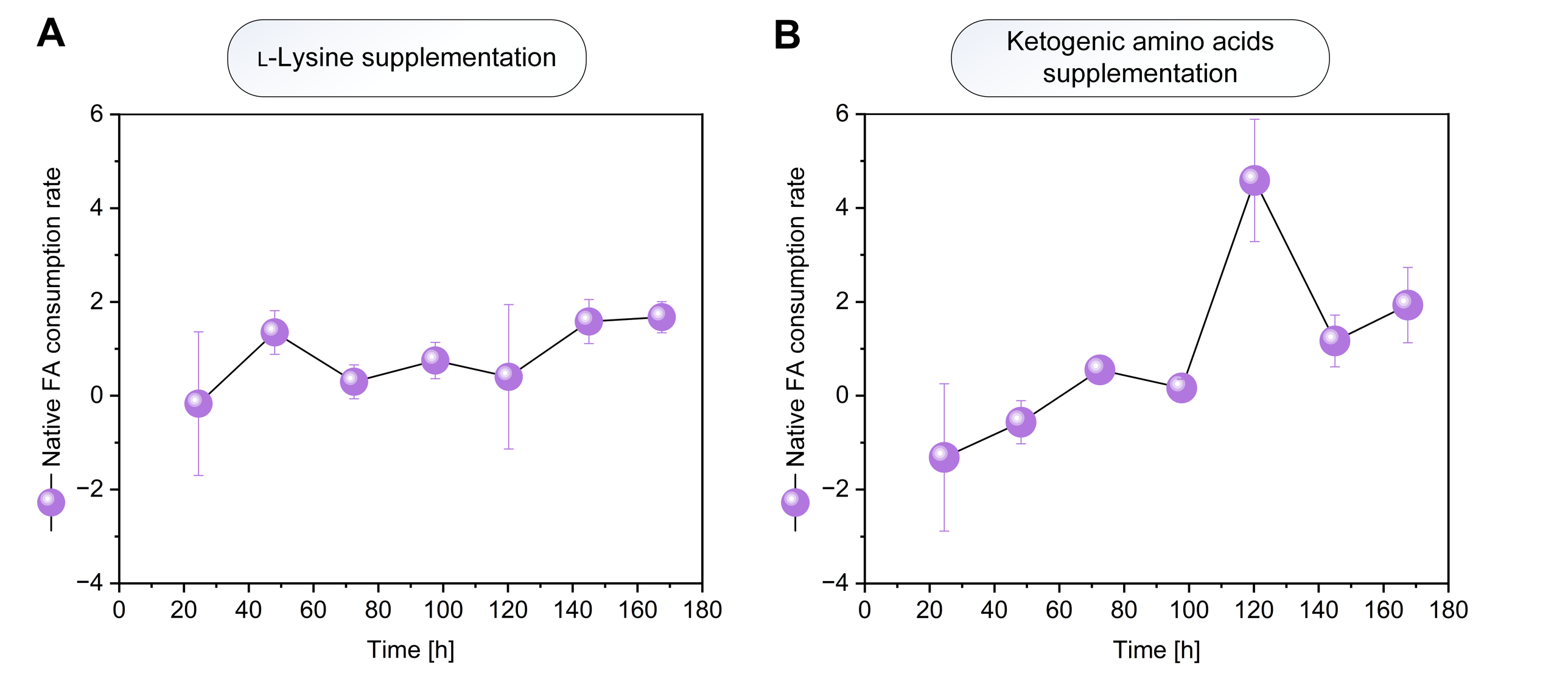


**Figure S3. Dynamics of the relative consumption rate of native fatty acids during glycerol-based cultivation.** Data refers to cultures with L-lysine supplementation (A) or ketogenic amino acid supplementation (B), compared to the non-supplemented glycerol cultivation process. Data are shown as a percentage of total fatty acid consumption. Data represent mean ± SD (n = 3).

###



**Figure S4. Volcano plots comparing differential gene expression between supplemented and non-supplemented conditions during the late stationary phase.** (A) L-lysine supplementation vs. control, (B) ketogenic amino acids supplementation vs. control. (C) Gene Ontology (GO) enrichment analysis using the Panther statistical overrepresentation tool, based on differentially expressed genes (DEGs) from (A), with an adjusted p-value < 0.05 and |log₂ fold change| ≥ 1. Plots show the proportion of DEGs in significantly enriched GO categories (orange for upregulated genes, blue for downregulated genes). Significantly enriched biological processes are indicated by asterisk (*FDR < 0.05, ** FDR < 0.01, *** FDR < 0.001). Data represent mean (n = 3).

**

**

**Figure S5. KEGG pathway enrichment analysis under amino acid supplementation.** Differentially expressed genes (DEGs) with an adjusted p-value < 0.05 and |log₂ (fold change) | ≥ 1 were analyzed for their association with enriched pathways. Enrichment plots compare gene expression profiles between supplemented and non-supplemented conditions during the early production phase: (A) L-lysine supplementation vs. control, (B) ketogenic amino acid supplementation vs. control. Pathway ratios are calculated as GeneRatio / BgRatio, representing the proportion of DEGs within a given pathway. Significant pathway enrichment is indicated as follows: * *P* adj < 0.05, ** *P* adj < 0.01, *** *P* adj < 0.001. Data represent mean (n = 3).

###
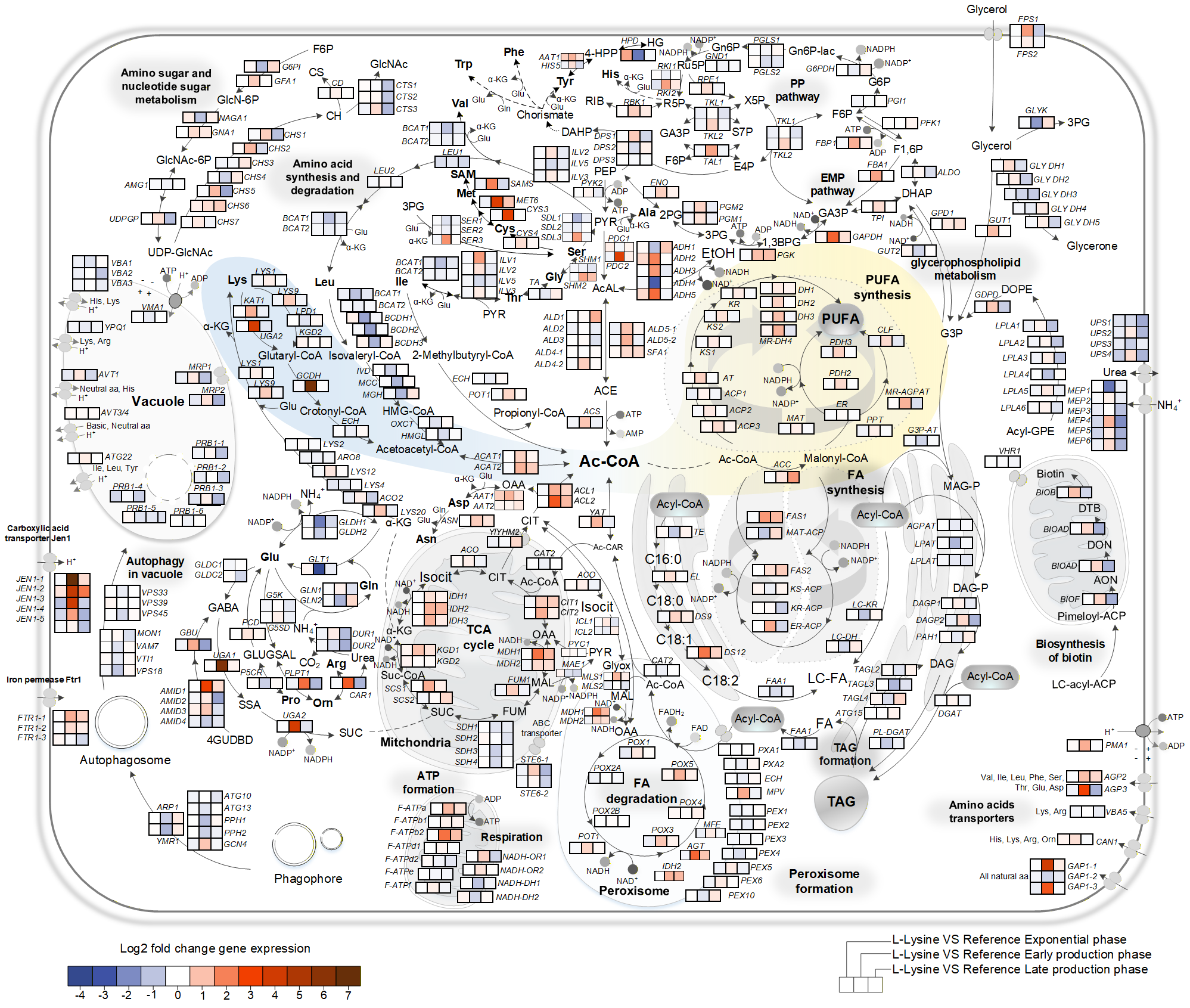


**Figure S6. Transcriptional dynamics in central metabolism and supporting pathways of *Y. lipolytica* Hyb6 expressing a myxobacterial PKS-like synthase for PUFA production from glycerol.** Gene expression was measured at three time points: exponential growth phase (10 h), early stationary phase (48 h), and late stationary phase (96 h) under L-lysine-supplemented conditions, compared to reference without supplementation. Data represent mean (n = 3). The 350 genes analyzed represent key metabolic pathways involved in glycerol utilization, citrate and acetate metabolism, the EMP pathway, the pentose phosphate pathway, the TCA cycle, CoA ester metabolism, amino acid metabolism, lipid synthesis and breakdown, PUFA formation, and other supporting pathways. Full gene lists and raw data are available at the Gene Expression Omnibus (GEO). Abbreviations: 1,3BPG: 1,3-Bisphosphoglyceric acid; 2PG: 2-Phosphoglycerate; 3PG: 3-Phosphoglycerate; 4GUDBD: 4-Guanidinobutanamide; 4GUDBUTN: 4-Guanidinobutanoate; 4HPP: 4-Hydroxyphenylpyruvate; AcAL: Acetaldehyde; Ac-CAR: Acetyl-carnitine; ACE: Acetate; Acyl-GPE: Acyl-sn-glycero-3-phosphoethanolamine; AON: 8-Amino-7-oxononanoate; CIT: Citrate; CH: Chitin; CS: Chitosan; DAG: Diacylglycerol; DAG-P: Diacylglycerol phosphate; DAHP: 2-Dehydro-3-deoxy-D-arabino-heptonate 7-phosphate; DHAP: Dihydroxyacetone phosphate; DON: 7,8-Diamino-nonanoate; DOPE: sn-Glycerol-3-phosphoethanolamine; DTB: Dethiobiotin; E4P: Erythrose 4-phosphate; F1,6P: Fructose 1,6-bisphosphate; F6P: Fructose 6-phosphate; FUM: Fumarate; G3P: Glycerol-3-phosphate; G6P: Glucose 6-phosphate; GA3P: Glyceraldehyde 3-phosphate; GABA: γ-Aminobutyric acid; GlcNAc: N-Acetylglucosamine; GlcN-6P: Glucosamine-6-phosphate; GlcNAc-6P: N-Acetylglucosamine-6-phosphate; GLUGSAL: L-Glutamate 5-semialdehyde; Gn6P: Gluconate 6-phosphate; Gn6P-lac: D-Glucono-1,5-lactone 6-phosphate; HG: Homogentisate; Isocit: Isocitrate; LC-FA: Long-chain fatty acids; MAG-P: Monoacylglycerol phosphate; MAL: Malate; OAA: Oxaloacetate; PEP: Phosphoenolpyruvate; PYR: Pyruvate; R5P: Ribose 5-phosphate; RIB: Ribose; Ru5P: Ribulose 5-phosphate; S7P: Sedoheptulose 7-phosphate; SSA: Succinate semialdehyde; SUC: Succinate; TAG: Triacylglycerol; UDP-GlcNAc: Uridine 5′-diphospho-N-acetylglucosamine; X5P: Xylulose 5-phosphate; α-KG: α-Ketoglutarate.

###
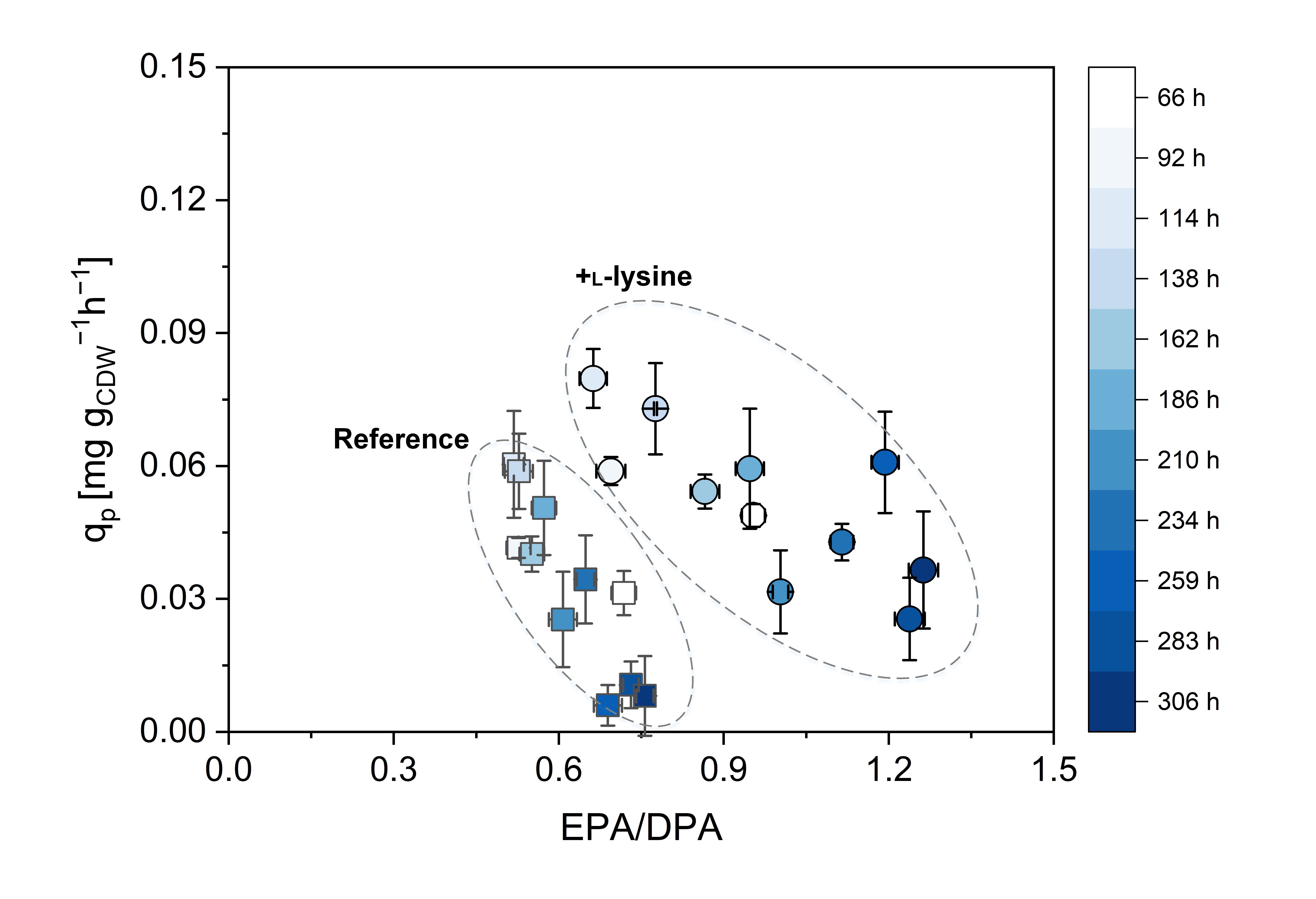


**Figure S7. Correlation between EPA/DPA ratio and specific product formation rate (qₚ) during fed-batch fermentations.** The EPA/DPA ratio is shown on the x-axis, with corresponding qₚ values on the y-axis. Both reference and L-lysine-supplemented conditions from Figure 8 are represented. The data highlight the relationship between the EPA/DPA ratio and product yield during the fermentation process. Data represent mean ± deviation (n = 2).
